# Balancing water conservation and health: do water-saving showerheads impact the microbes we breathe in during showering?

**DOI:** 10.3389/frmbi.2024.1416055

**Published:** 2024-07-15

**Authors:** Sarah Pitell, Cheolwoon Woo, Evan Trump, Sarah-Jane Haig

**Affiliations:** ^1^ Department of Civil and Environmental Engineering, University of Pittsburgh, Pittsburgh, PA, United States; ^2^ School of Public Health, University of Pittsburgh, Pittsburgh, PA, United States

**Keywords:** shower aerosol, bioaerosol, water saving devices, low-flow showerheads, microbiome, *Legionella*, NTM, biofilm

## Abstract

Low-flow showerheads offer consumers economic and water-saving benefits, yet their use may inadvertently affect the microbial content of produced water and water-associated aerosols. This study aimed to compare the abundance and microbial composition of bacteria in shower water and associated respirable aerosols produced by various low flow rate (1, 1.5, and 1.8 gpm) showerheads. Our findings indicate that the lowest-flow showerhead produces water with lower total microbial and opportunistic bacterial pathogen densities compared to higher low flow rate counterparts. However, microbiome analysis revealed that 1.8 gpm flow rate showerheads exhibit reduced abundance of Gram-negative organisms and common biofilm-forming organisms, suggesting potentially lower pathogenicity compared to 1 and 1.5 gpm low-flow showerheads. Additionally, the number of respirable aerosols produced by showerheads as well as the partitioning of certain microorganisms from the water to aerosol phases was negatively correlated with flow rate, suggesting that there may be increasing exposure potential to pathogenic bioaerosols when using a 1gpm showerhead compared to a 1.8 gpm showerhead. However, the 1.5 gpm showerhead seemed to balance microbial partitioning, aerosol generation, and water conservation. Moreover, the microbial composition of aerosols produced from shower water was more influenced by the age of the showerhead than the flow rate, highlighting the significance of biofilm formation on the microbial community. Overall, our findings underscore the importance of evaluating the microbial risk associated with low-flow showerheads using multiple metrics in both water and aerosols, and dynamically assessing this over time, to ensure accurate future risk assessment.

## Introduction

Low-flow water fixtures have been increasingly adopted to conserve water and energy, with many proponents citing the economic and environmental benefits that can be achieved through these kinds of fixtures (Thornton and Tanner, 2010; Abu-Bakar et al., 2021). For instance, U.S. households can save up to 2,700 gallons of water annually by using showerheads endorsed by the U.S. Environmental Protection Agency (U.S. EPA, 2014), and high-efficiency appliances in households have led to changes in water consumption habits and lower bills, contributing to tangible water savings (Lee and Tansel, 2013). Aside from the economic implications, in some societies, it might be taken for granted that conserving water and energy through the enhancement or replacement of existing facilities is a necessity for the future. Due to global population growth and climate change, the demand for potable water has increased unprecedentedly, driving agencies such as the U.S. EPA to invest in research and policy change focused on redesigning or retrofitting residential point of use water fixtures such as toilets, showers, and washing machines to save water and energy (Mayer et al., 1999; Willis et al., 2010; Wong et al., 2016). For example, the WaterSense program, sponsored by the U.S. EPA, offers consumers an easy method to recognize products, homes, and services that are water-efficient (U.S. EPA, 2024). As an illustration, the specifications for WaterSense showerheads establish a maximum flow rate of 2.0 gpm at pressures up to 80 psi, which represents 20% more efficiency compared to the federal standard of 2.5 gpm. While these conservation-related improvements are beneficial to consumers and the environment alike, there may be unintended consequences from a public health perspective due to the mechanisms deployed to reduce water use that have not been evaluated.

Low-flow showerheads (≤2 gpm) reduce water usage by employing atomization technology, which produces smaller water droplet sizes that evaporate or break apart and aerosolize more quickly than droplets generated by conventional showerheads (2.5 gpm) (Niculita-Hirzel et al., 2021). In addition, low-flow showerheads eject more respirable aerosols (less than 10 µm – capable or reaching deep into the respiratory system) than conventional showerheads (Estrada-Perez et al., 2018; Niculita-Hirzel et al., 2021), which is due to the adjustment of showerhead characteristics to compensate for the lower flow rate: larger spray angle, higher water pressure, reduced number of nozzles, and increased nozzle diameter, which in turn may cause more aerosols (Niculita-Hirzel et al., 2021). The properties of these aerosols may be impacted by the bulk water they are generated from, which can be concerning if there are respirable hazards present. Despite verification that low-flow showerheads provide similar consumer satisfaction (Thornton and Tanner, 2010; U.S. EPA, 2010; Williams et al., 2013) and water quality to conventional showerheads, the relationship and impacts between the specific low-flow, flow rate, and the number and size of aerosols generated and whether these aerosols contain organisms capable of causing infection has not been investigated.

Previous work has shown that the surfaces within building plumbing systems including shower hoses offer a conducive environment for the formation of biofilms, thereby enabling a diverse range of microorganisms, including potential pathogens, to survive and proliferate (Angenent et al., 2005; Feazel et al., 2009; Craun et al., 2010; Moritz et al., 2010; Proctor et al., 2022). Further, another study on initial colonization in virgin plumbing materials found that DW biofilms change over time, with marked differences occurring around 30 days of continuous use (Morvay et al., 2011). In particular, Drinking Water Pathogens which predominately infect the Immunocompromised (DWPIs) such as *Legionella pneumophila* and nontuberculous mycobacteria (NTM) reside in building plumbing biofilms and are responsible for costing the U.S. healthcare system $2.39 billion annually in direct healthcare costs (Collier et al., 2021; Proctor et al., 2022), and the incidence of related outbreaks is continuing to increase (Kunz, 2024). One potential route for DWPI infection is through the inhalation of aerosols produced during daily activities such as showering, using hot tubs, therapy pools, and recreational swimming (Bollin et al., 1985; Angenent et al., 2005; Falkinham et al., 2015; Proctor et al., 2022), therefore an increase in aerosol generation due to changes in shower system design (e.g., reduction in flow rate) could lead to the elevation of respirable aerosols containing DWPIs, thus increasing the risks of DWPI infection. Despite a previous study finding DWPIs such as *Legionella* enriched in respirable aerosols (less than 10 µm) generated by a 1.5 gpm low-flow showerhead (Estrada-Perez et al., 2018) it remains unknown how differing low flow rates influence potential DWPI exposure risk and how other microbiome members are impacted. It is expected that due to the greater number of aerosols generated from these low-flow showerheads, there will be larger incidence of microbial aerosolization, including DWPIs.

In today’s market, there are many kinds of low-flow showerheads available leaving consumers to choose solely based on economic and energy efficacy preferences (Horne, 2009). However, making choices based solely on these factors could potentially harm users’ respiratory health due to the increased aerosol production of low-flow showerheads. Therefore, based on this, a thorough examination of the shower-associated microbes present in both the water and aerosols generated by low-flow showerheads is needed to ascertain whether differences in low flow rates impact potential consumer risk. In this study, weekly shower water and shower water-associated aerosols samples generated from nine showerheads (three different low flow rates in triplicate) operating in a full-scale shower laboratory to simulate real-world shower dynamics were collected. The concentration of total bacteria, DWPIs and the microbial community composition were assessed in all samples, with water chemistry aspects and total aerosol generation dynamics also measured.

## Materials and methods

### Full-scale shower laboratory

Sampling occurred in the full-scale INHALE shower laboratory as previously described (Pitell and Haig, 2023). Briefly, the INHALE laboratory contains three shower stalls, each with three commonly purchased acrylonitrile butadiene styrene plastic (ABS) low-flow showerheads with flow rates of 1, 1.5, and 1.8 gpm in each stall (n=9 showerhead outlets). Each stall was supplied by hot water from a domestic-scale water heater that receives its input water from the building drinking water supply. The INHALE shower laboratory was operated to simulate a domestic bathroom: the water exiting the showerhead was set to be 40°, the average showering temperature of an American, and each showerhead was flushed every day for 8 minutes, the average showering time for an American (Wilkes et al., 2005). Each shower stall door contained a small hole 154 cm from the bottom of the shower stall to facilitate aerosol collect from the average respiratory zone of Americans (McDowell et al., 2009). The sampling campaign spanned eight weeks where sampling events occurred weekly with a two-week gap between weeks 4 and 5 to simulate longer stagnation.

### Aerosols and water samples

Three different types of samples were collected for each showerhead over the six sampling events. Aerosol particle number and diameter were collected with the AeroTrak Handheld Particle Counter 9306-03 (TSI, Inc., Shoreview, MN, USA) in bins of 0.3–0.5, 0.5–0.7, 0.7–1, 1–2, and 2–5 µm. The particle counter was programmed to take a reading once every 3 seconds at a flow rate of 2.83 liters per minute. The particle counter was run for 30 minutes for each showerhead: 5 minutes before turning on the showerhead to assess background particulates, 20 minutes while the shower was running, and 5 minutes after turning it off to monitor particle dissipation. A pause of 20 minutes was implemented between the measurements from different showerheads, permitting the laboratory conditions to revert to the initial temperature and relative humidity and allow particle concentrations to reach room baseline values.

Airborne bacterial particles were collected with a Series 110A Spot Sampler™ aerosol particle collector (Aerosol Devices, Inc., Fort Collins, CO, USA) with the addition of a SCC1.829 cyclone (Mesa Laboratories, Inc., Lakewood, CO, USA) that allowed for collection of respirable aerosols (<10 µm in diameter). The particle collector was run for 40 minutes while each showerhead was running at an aerosol collection flow rate of 1.5 liters per minute and the airborne particles were collected in 0.5 mL of phosphate-buffered saline (PBS) following the previously described methods (Nieto-Caballero et al., 2019; Pitell and Haig, 2023). Additionally, controls were conducted in the form of one background control per week where the collector was run without the shower turned on, and one HEPA control where the collector was fed air that had already passed through a HEPA filter. After sampling, PBS was transferred to a sterilized 2 mL tube and stored at −20°C until subsequent analysis.

Composite water samples from each showerhead were collected over the course of 8 minutes, totaling 1.3 L of shower water in a sterile Nalgene bottle. 1 L of the water was filter concentrated onto a 0.2 µm polycarbonate filter (Millipore Sigma, Burlington, MA, USA) for molecular analysis and preserved at −20°C until subsequent analysis. Additionally, field controls where deionized water was processed onsite identically to an experimental sample and filter controls where an unused filter was collected as a sample were run during each sampling event. The remaining water was used to assess the shower water quality. Temperature and pH were measured on-site using a temperature meter (Hanna Instruments, Inc., Woonsocket, RI, USA) and a portable pH probe (Mettler-Toledo, LLC, Columbus, OH, USA), respectively. Free and total chlorine were measured at the time of collection using the DPD method (Hach Company, 2022) on a portable DR900 spectrophotometer (Hach Company, Loveland, CO, USA). Total and dissolved iron, lead, copper, silver, calcium, and magnesium concentration were determined using inductively coupled plasma mass spectrometry with a NexION 300 ICP-MS (PerkinElmer, Inc., Waltham, MA, USA). Prior to analysis, all dissolved metal samples were prepared by passing water through a 0.45 µm nylon syringe filter (Thermo Fisher Scientific, Inc., Waltham, MA, USA) primed with 5 mL of sample.

### DNA extraction

DNA was extracted from the collected water sample filters, PBS containing air samples, field, filter and extraction control samples using the Fast Spin DNA Extraction kit (MP Biomedicals, Irvine, CA, USA) following the optimized protocol outlined (Haig et al., 2018). The extracted DNA was stored at −20°C until subsequent analysis.

### Droplet digital PCR

The concentration of total bacteria, *L. pneumophila*, and NTM were determined by droplet digital PCR (ddPCR) following the method outlined in the previous study (Pitell and Haig, 2023). Briefly, the 16S rRNA gene was targeted for determining the total bacteria concentration, and each bacteria’s specific gene was targeted respectively (**Supplementary Table 1**) for quantification on a QX200 ddPCR System (Bio-Rad Laboratories, Inc., Hercules, CA, USA). The ddPCR samples were cycled with the following conditions: initial denaturation at 95°C for 5 min, 45 cycles of 30 s at 95°C, 60 s at 57°C, and 60 s at 72°C, and polymerase inactivation by 5 min at 4°C and 5 min at 90°C. Data was analyzed using Quantasoft version 1.0.596 following the previously described methods (Lievens et al., 2016; Pitell and Haig, 2023). All samples were analyzed in duplicate along with negative controls (field blanks, extraction blanks, and ddPCR blanks of molecular grade water as the template) and gblock positive controls of each amplicon (Integrated DNA Technologies, Inc., Coralville, IA, USA).

### 16S rRNA gene amplicon sequencing

The overall microbial community in each sample was assessed by 16S rRNA gene amplicon sequencing, which was conducted on a HiSeq 2500 system (Illumina, Inc., San Diego, CA, USA). Both library preparation and sequencing were carried out by Argonne National Laboratory (Lemont, IL, USA) following the Illumina Earth Microbiome Protocol (Caporaso et al., 2012). Sequencing data processing and quality controls were performed on QIIME2 version 2020.2 (Bolyen et al., 2019). Reads were assigned to operational taxonomic units (OTUs) using a 97% cutoff and taxonomic assignment for each OTU was performed against the SILVA version 132.5 reference database (Quast et al., 2013). In addition, functional abundances were predicted by PICRUSt2 (Douglas et al., 2020). A total of 5,968,976 high-quality sequence reads were obtained from a total of 104 samples, divided into 1,157,322 reads from 53 air samples and 4,811,654 reads from 51 water samples (**Supplementary Table 2**). A total of 54 pairs of samples were collected during sampling, but one air sample and three water samples were excluded from the 16S rRNA gene amplicon sequencing because insufficient amplification was achieved.

### Statistical analysis

Statistically significant differences in the absolute number of aerosols and the absolute densities of targeted bacteria in air and water samples based on showerhead flow rate and showerhead age were determined using Wilcoxon rank-sum and/or Kruskal-Wallis tests with continuity corrections in R version 4.3.2. Significant differences in the aerosol partitioning percentages were determined using a one-way analysis of variance (ANOVA) test. Statistical analysis on the sequencing results was conducted using the phyloseq (McMurdie and Holmes, 2013) and the vegan (Oksanen, 2010) packages. Bacterial diversity within (α diversity) and between samples (β diversity) were analyzed. In terms of α diversity, the Chao1 richness estimator and the Shannon index were considered to estimate bacterial richness and diversity within each sample, respectively. To compare the α diversity metrics, Wilcoxon rank-sum and/or Kruskal-Wallis tests were conducted. To assess the β diversity, Bray-Curtis dissimilarity and Jaccard index were computed to identify differences in bacterial structure and membership between samples, respectively. Differences in bacterial community composition due to sample types (air and water samples) or showerhead features (flow rate and days since installation) were assessed using permutational multivariate analysis of variance (PERMANOVA). Predicted differences in the functional profiles, determined by the PICRUSt2 (Douglas et al., 2020), were analyzed with the ggpicrust2 package (Yang et al., 2023). Additionally, the differences between sample types and showerhead features were determined through LinDA (Zhou et al., 2022) with significance adjusted for multiple comparisons using the Benjamini-Hochberg false discovery rate correction.

## Results

### Water-conserving showerheads decreased overall microbial concentrations in shower water

**Figure 1:**
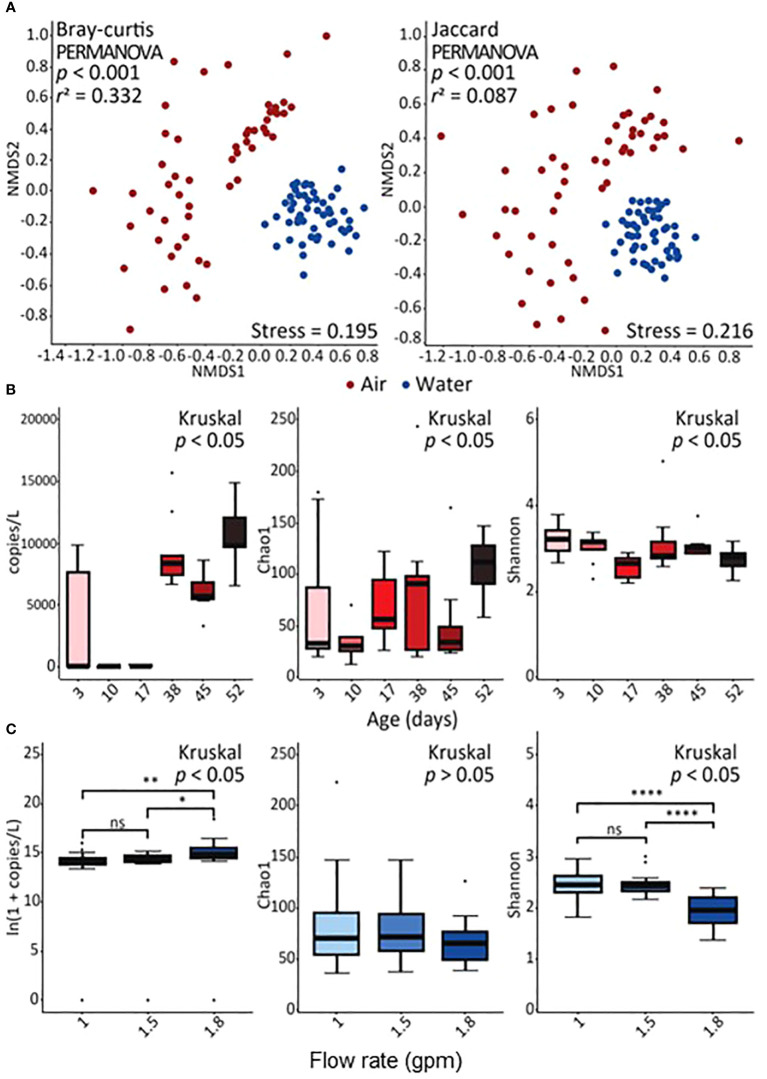
Diversity of identified bacteria. The results shown are based on the operational taxonomic units (OTUs). **(A)** Non-metric multidimensional scaling (NMDS) plots show the structure and membership of bacterial OTUs represented by the Bray–Curtis dissimilarity and Jaccard index, respectively. **(B)** The concentration of bacteria in air samples over time and the comparison of richness and diversity of bacterial OTUs in air samples over time estimated by the Chao1 estimator and Shannon index, respectively. **(C)** The concentration of bacteria in water samples by flow rate and comparison of richness and diversity of bacterial OTUs in water samples by flow rate estimated by the Chao1 estimator and Shannon index, respectively. In panel **(C)**, one asterisk (*), two asterisks (**), and four asterisks (****) represent *p* < 0.05, *p* < 0.01, and *p* < 0.0001, respectively, by the *post hoc* Wilcoxon rank-sum test. The abbreviation “ns” represents no statistical difference.

Although chemical water quality was not significantly affected by showerhead flow rate (**Supplementary Tables 5**, **6**), absolute abundances determined via ddPCR analysis revealed that flow rate had minimal impacts on DWPIs and total bacteria in water or associated aerosols (**Figure 1**; **Supplementary Figures 1**–**3**). Microbial densities were significantly greater in water samples than air samples (**Supplementary Figures 1**–**3**), as anticipated given the considerable disparity in densities between the two sample types (averaging 4.29 × 10^6^ gene copies/L in water samples versus 4.81 × 10^3^ gene copies/L in air samples). In the water samples, low flow showerheads with the flow rate of 1.8 gpm had the largest total bacteria and NTM abundances of the three flow rates tested (**Figure 1**; **Supplementary Figures 1**, **2**). However, for DWPI abundance, few differences were found between the different showerhead flow rates, with NTM abundance only found to be statistically different between the 1.5 gpm head and 1.8 gpm head. No significant differences in *L. pneumophila* abundances were observed between any flow rate likely due to its infrequent detection (43% and 41% of water and air samples, respectively; **Supplementary Table 4**). There were no differences between *L. pneumophila*, NTM, or total bacteria abundances by flow rate for the air samples.

### Aerosolization partitioning is flow rate and DWPI dependent

**Figure 2:**
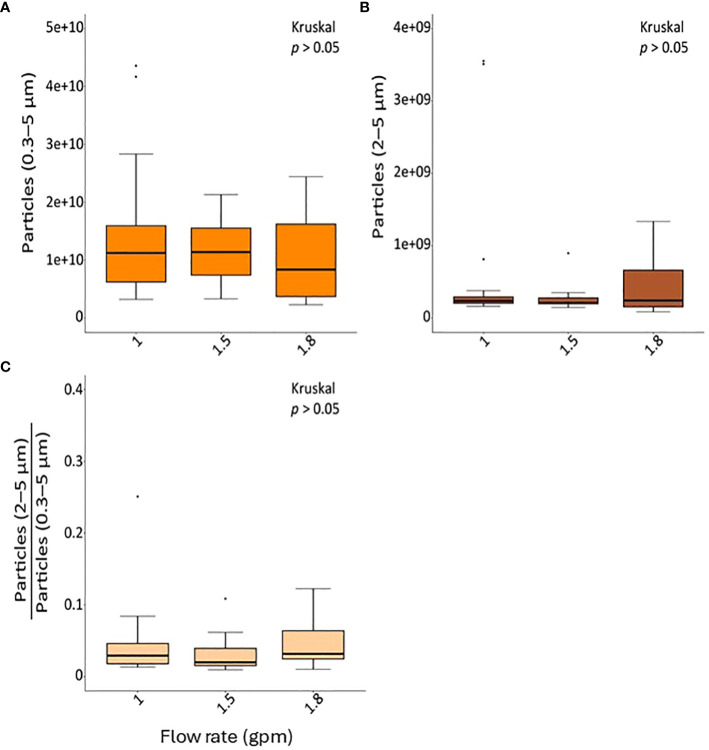
The concentration of particles generated from the showerheads according to each flow rate. **(A)** The concentration of total particles with diameters 0.3–5 µm, **(B)** the concentration of particles with diameters 2–5 µm, and **(C)** the ratio of particles with diameters 2–5 µm compared to total particles are shown.

Both total inhalable aerosol counts (0.3–5 µm) and bio-respirable aerosol counts that may contain bacteria (2–5 µm) did not significantly differ by showerhead flow rate during the sampling period (**Figure 2**). On average 1 × 10^9^ inhalable and 3.7 × 10^7^ bio-respirable aerosols were produced during the 30 min of collection equating to approximately 2.7 × 10^8^ particles and 9.8 × 10^6^ particles in the average 8-minute shower, respectively.

Partitioning behavior of DWPIs and total bacteria (the proportion of microorganism recovered in the aerosol phase vs. the water phase) was also evaluated to assess if there was an increased ratio of bacteria transitioning from the air to the water phase that could possibly be inhaled during a showering event (**Table 1**). *L. pneumophila*, NTM, and total bacteria all had different partitioning behavior from each other, and they varied with showerhead flow rate. More specifically, the 1 gpm showerhead samples had the most partitioning of NTM and overall microbial densities, and *L. pneumophila* had the higher partitioning in samples taken from the 1.8 gpm showerhead.

**Table 1:** Average aerosolization partitioning percentages for DWPIs and total bacteria.

	Average aerosolization partitioning percentage ± standard deviation (%)			*p-*value from ANOVA
	Low-flow showerhead flow rate			
	1 gpm	1.5 gpm	1.8 gpm	
**Total bacteria**	0.39 ± 0.42	0.22 ± 0.34	0.12 ± 0.17	0.01
***L. pneumophila***	2.93 ± 2.06	1.01 ± 1.62	9.1 ± 9.9	0.05
**Nontuberculous mycobacteria**	0.005 ± 0.008	0.002 ± 0.002	0.002 ± 0.002	0.04

### Microbial community and functionality were affected by showerhead age and flow rate

The overall microbial community structure (alpha and beta diversity) was significantly different in air and water samples (**Figure 1A**), with distinct differences further occurring between the different flow rates. Significant differences were identified in β diversity, such as structure (Bray–Curtis dissimilarity) and membership (Jaccard index) of bacterial OTUs, between the sample types (*r* ^2^ = 0.332 and 0.087, respectively, *p* < 0.001; PERMANOVA) (**Figure 1A**). In particular, OTU diversity was greater in aerosol than water samples, and diversity in water samples decreased as a function of flow rate while diversity in air samples differed based on showerhead age (**Figures 1B, C**; **Supplementary Table 3**).

**Figure 3:**
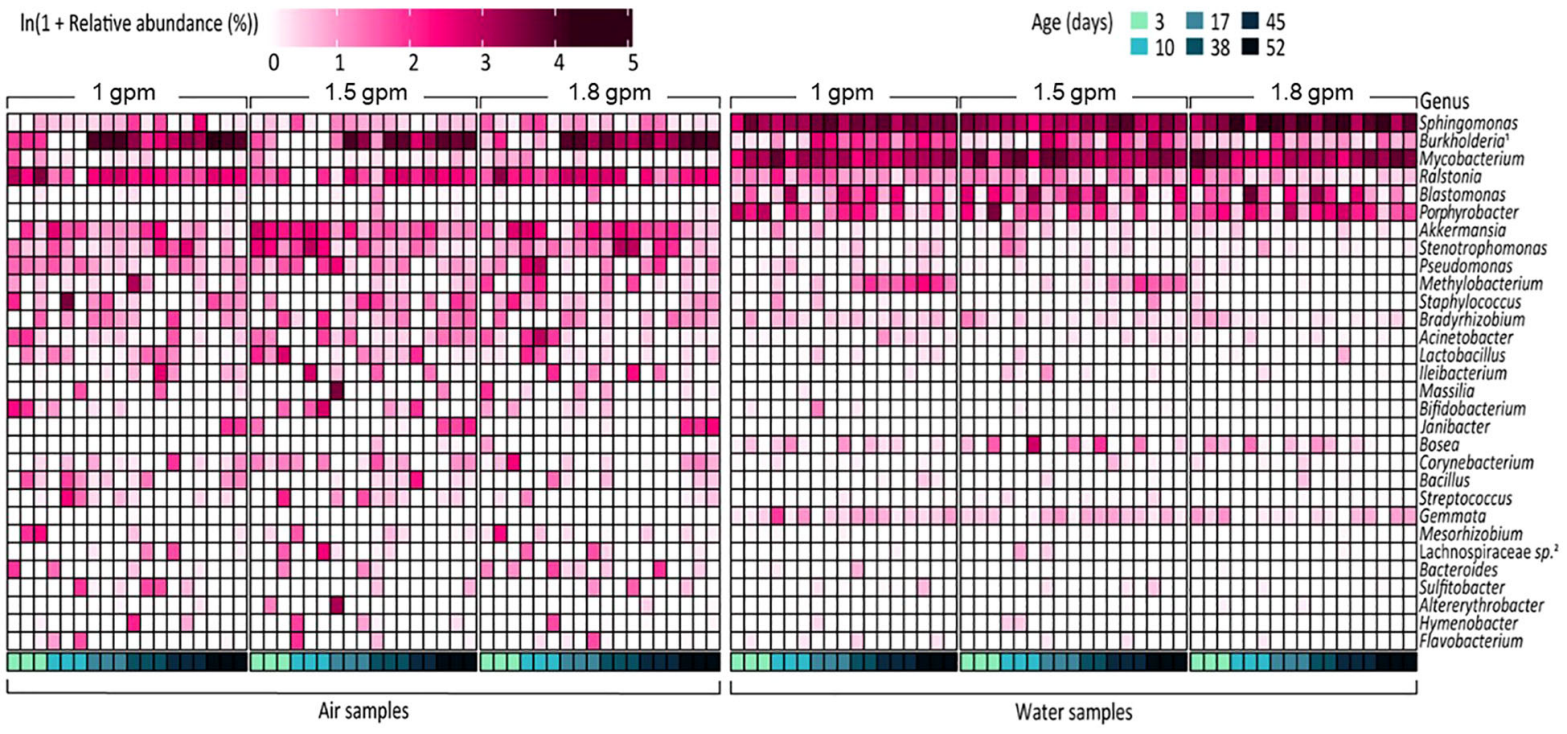
Relative abundance of the 30 most abundant bacterial genera in all samples. In the figure, *Burkholderia* ^1^ and Lachnospiraceae sp.^2^ refer to *Burkholderia-Caballeronia-Paraburkholderia* and Lachnospiraceae NK4A136 group, respectively.

The dominant microbial community membership also varied in aerosol and water samples (**Figure 3**; **Supplementary Figure 4**), with the majority of the relative abundance of water samples being composed of a few genera (e.g., *Sphingomonas, Burkholderia* sp.*, Mycobacterium*, and *Prophyrbacter)* whereas aerosols samples showed more taxonomic diversity in its core microbiome. Common core/shared microbiome members of both water and aerosols samples were *Sphingomonas, Burkholderia-Caballeronia-Paraburkholderia*, and *Ralstonia* which are microorganisms commonly associated with drinking water (Pitell and Haig, 2023), and known to contain DWPIs linked to nosocomial infections (Ryan and Adley, 2010, 2014; Proctor et al., 2022). In the water samples, the relative abundances of *Sphingomonas* and *Ralstonia* appear to be flow dependent, with *Ralstonia* being more consistently detected in higher abundances in the 1 gpm showerhead samples and *Sphingomonas* being more enriched in the 1.8 gpm showerhead samples. In air samples, no specific taxa showed clear correlations with flow rate, but complex temporal dynamics were observed. This observation is exemplified by Burkholderia-Caballeronia-Paraburkholderia. *Burkholderia* spp. constituted a significant proportion of the total relative abundance in the air samples, regardless of showerhead flow rate or sampling time (23/8% ± 21.7%). Nonetheless, its establishment dynamics in both water and air samples were intricate, showing a decrease in relative abundance from the day of installation until 38 days of continuous use, followed by a dramatic increase and eventual dominance after 38 days, which is in agreement with previously reported biofilm dynamics (Morvay et al., 2011). Similar dynamics were observed in the relative abundance of *Methylobacterium* in samples from the 1 gpm and 1.5 gpm showerheads.

**Figure 4:**
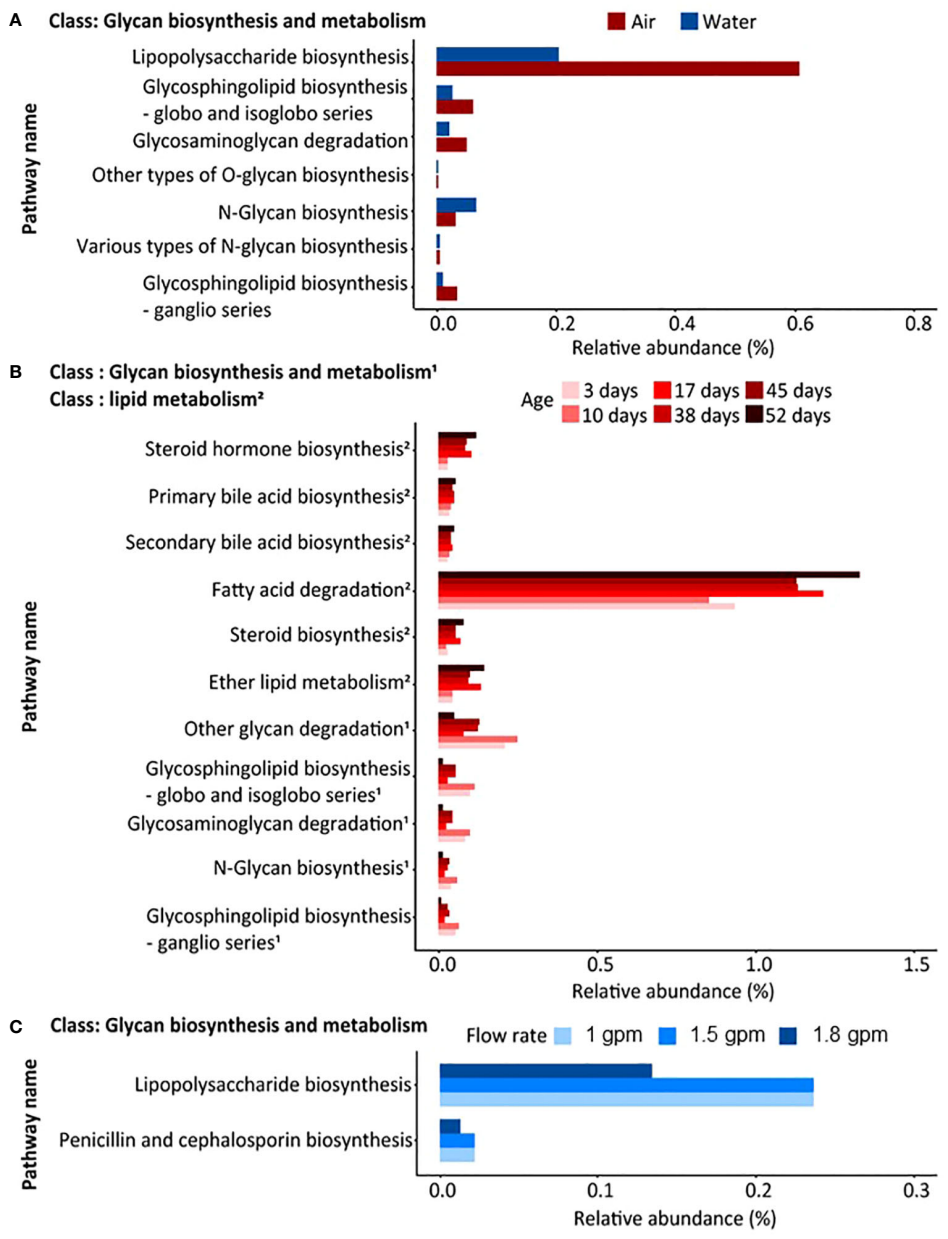
Statistically significant functional features, obtained from PICRUSt2, with p-values less than 0.05 confirmed by differential abundance and *post hoc* analysis. The names of pathways and the upper category of each pathway, i.e., class, are shown. The significantly different functional features **(A)** between air and water samples, **(B)** in air samples by age factor, and **(C)** in water samples by flow rate are shown. In panel B, two different classes are marked as 1 and 2.

Predictive functional composition analysis using PICRUSt2 revealed significant variations between sample types (air and water samples), with distinct dynamics observed based on shower-related factors such as showerhead age (days since installation) and flow rate (**Figure 4**). A total of 78 statistical differences in metabolic functional features were identified when comparing air and water samples (**Supplementary Table 7**). Specifically, 35 statistical differences were observed in air samples when categorized by showerhead age (**Supplementary Table 8**), while three statistical differences were detected in water samples when analyzed by flow rate (**Supplementary Table 9**). Notably, glycan biosynthesis and metabolism exhibited prominent differences between air and water samples, with lipopolysaccharide (LPS) biosynthesis being more abundant in air samples compared to water samples (**Figure 4A**). Further analysis revealed that functional features showing statistically significant differences were influenced differently by shower factors based on the type of sample (air or water). Air samples exhibited a notable impact from the age of the shower system, with glycan biosynthesis and metabolism being more abundant shortly after installation and lipid metabolism becoming more prominent after 52 days of operation (**Figure 4B**). Conversely, water samples were significantly affected by flow rate, with a decrease in flow rate correlating with higher abundances of glycan biosynthesis and metabolism (**Figure 4C**). Overall many of the microbial genera and predictive functional characteristics influenced by showerhead flow rate have been associated with increased asthma and rhinitis attacks (Fu et al., 2021) suggesting that showerhead flow rate may play a currently unknown role in allergic respiratory disease. It should, however, be stressed that in order to link members of the shower aerosol microbiome to human health outcomes, bacterial species, strain, and virulence data are needed.

## Discussion

In this study, we examined the abundance and composition of bacteria in both shower water and respirable aerosols (particles less than 10 µm in diameter) emitted by showerheads with varying low-flow rates (1 gpm, 1.5 gpm, and 1.8 gpm) over an 8-week period within a full-scale shower laboratory. Additionally, we assessed the concentration of total respirable airborne particles produced at each flow rate. Our findings reveal that the absolute abundance of DWPIs remained unaffected by flow rate in either phase, except for NTM, which exhibited a significant elevation in water samples from the 1.8 gpm showerheads (**Supplementary Figure 2**). Moreover, we observed an increase in total bacteria in water samples relative to increasing flow rate. Notably, flow rate influenced the bacterial community composition in shower water, while showerhead age impacted the microbiome composition of shower aerosols. Examination of DWPI and total bacteria partitioning demonstrated a variation in aerosolization behavior based on flow rate, with 1.5 gpm showing the lowest partitioning while still conserving water. Additionally, the lower flow rate showerheads (1.0 gpm) produced more particles within the diameter range of 2–5 µm, along with a higher proportion of fine particles ranging from 0.3 to 2 µm (**Figure 2**), which may provide more opportunities for microorganisms to aerosolize. These findings align with previous studies which have shown the propensity for low-flow showerheads to increase the amount of respirable particles capable of containing bacteria compared to standard flow showerheads (Zhou et al., 2007; Estrada-Perez et al., 2018; Niculita-Hirzel et al., 2021. Given the size range of bacterial cells (2 to 10 µm) (Woo and Yamamoto, 2020), and the fact that this study and previous work (Perkins et al., 2009; Estrada-Perez et al., 2018) have detected DWPIs and other viable or potentially pathogenic bacteria in both shower water and aerosols produced from low-flow showerheads, a comprehensive microbial risk assessment associated with flow rate in shower systems is imperative to fully understand microbial exposure risk.

### Temporal variations in the bacterial dynamics of shower system may be caused by biofilm formation

Throughout the 8-week sampling period, it is assumed that a biofilm was developed inside the shower system, and because of this, the dynamics of the bacterial communities in both phases were observed to change over time. The surfaces of building plumbing systems are known to promote biofilm formation, allowing a variety of microorganisms including potential pathogens, to persist and grow within the pipes (Angenent et al., 2005; Feazel et al., 2009; Craun et al., 2010; Moritz et al., 2010; Proctor et al., 2022). Water samples, representing the overall bacterial communities supplied to the shower system, showed no significant changes in concentration, richness, and evenness during the 8-week sampling period (**Supplementary Figure 1C**, **Supplementary Table 3**). In contrast, aerosolized bacterial communities from showerheads showed increased total bacterial concentration and richness, coupled with a decrease in evenness over time (**Figure 1B**), suggesting a shift towards a more homogeneous bacterial composition, driven by the proliferation of some bacterial groups. This is supported by the observed increase in the prevalence of specific bacterial taxa after 38 days of continuous use, for example, *Burkholderia-Caballeronia-Paraburkholderia* and *Janibacter* (**Figure 3**). These microorganisms are known to thrive in developing biofilms, so their elevated relative abundance in the samples may suggest that detachment from a proliferated biofilm is occurring (Huber et al., 2002; Bragonzi et al., 2012). Surprisingly OTU diversity was greater in aerosol samples than in water samples regardless of flow rate or showerhead age, something likely attributed to the selective and dynamic nature of the aerosolization process and biofilm contributions. environmental differences, likely due to the aerosolization process, which selectively enriches bacteria that can survive in air. More specifically, the timing and composition of biofilm detachment from shower surfaces is unknown, but it is possible that many of the organisms which resided within the plumbing biofilm are more aerosolizable and hence contribute to the increased diversity observed in aerosols. In addition, although environmental conditions in the shower lab were controlled as much as possible it is possible that differences in air temperature and humidity influenced the aerosolization process as has been observed in other aerosol microbiome studies, but more work must be done to understand these effects. In addition to taxonomic changes, potential functional pathway shifts from glycan biosynthesis and metabolism dominance prior to 38 days to greater lipid metabolism after 38 days, which is consistent with current understanding of biofilm development (Dubois-Brissonnet et al., 2016) (**Figure 4B**). Interestingly, LPS biosynthesis was significantly higher in air samples than water samples (**Figure 4A**) suggesting that either these microorganisms were primed to synthesize more LPS because they were previously part of the biofilm, or that high LPS synthesis is a selecting parameter for aerosolization. The LPS, mainly known as one of the main cell surface structures of Gram-negative bacteria, is generally considered to play a role in allowing Gram-negative bacteria to adhere to surfaces during the early stages of biofilm formation (Donlan, 2002; Ruhal and Kataria, 2021). Therefore, it is likely that over time, a biofilm was developed within the shower system that likely contributed to the microbial characteristics of the shower water and associated aerosols.

The diverse microbial taxonomic compositions observed in our study is constant with previous DW building plumbing microbiome studies, with bacterial genera such as *Methylobacterium*, *Blastomonas*, *Bradyrhizobium, Bacillus*, *Mycobacterium*, *Pseudomonas*, *Legionella, Sphingomonas*, *Streptococcus*, *Stenotrophomonas*, and *Staphylococcus*, being abundant (Angenent et al., 2005; Feazel et al., 2009; Craun et al., 2010; Moritz et al., 2010; Proctor et al., 2016, 2018, 2022; Gebert et al., 2018). Moreover, absolute concentrations of DWPI-containing taxa, including NTM and *L. pneumophila*, were consistently detected in water samples from this study (**Figure 3**; **Supplementary Figures 2**, **3**), albeit at lower densities compared to findings in other studies (Feazel et al., 2009; Gebert et al., 2018). Discrepancies in absolute DWPI density may arise from variations in sample collection and DNA extraction procedures between our study and others. Notably, previous studies were conducted in more established systems, suggesting that higher DWPI abundances may be associated with mature biofilms. Although our study’s shower system biofilm is 5 years old and presumed immature compared to residential showers, it remains plausible that DWPIs could proliferate within the biofilm over time, as observed in prior studies (Feazel et al., 2009; Gebert et al., 2018). Consistent with water samples, these bacterial genera were also present in aerosol samples, with the *Burkholderia-Caballeronia-Paraburkholderia* group being particularly prominent (**Figure 3**). Interestingly, *Burkholderia’s* relative abundance in air samples declined over time, which contrasts with its persistence in biofilms in a recent water system study (Chen et al., 2023). Absolute quantification of NTM and *L. pneumophila* revealed low concentrations in aerosol samples (**Figure 3**; **Supplementary Figures 2**, **3**), which aligns with previous studies conducted in our laboratory (Pitell and Haig, 2023). However, despite other studies finding NTM as a readily aerosolizable genus (Falkinham, 1996; Estrada-Perez et al., 2018; Shen et al., 2022) due to its unique cell membrane structure (Dowdell et al., 2019) our study did not observe this, possibly due to methodological differences in our study, such as size exclusion during sampling, instrumentation used and sample collection time. Additionally, NTM aerosolization patterns may differ between older plumbing systems (Falkinham, 1996; Estrada-Perez et al., 2018; Shen et al., 2022) and our 5-year-old INHALE laboratory, suggesting that prolonged shower operation periods may be required for significant DWPI aerosolization to occur.

### Impact of flow rate on the bacterial communities of shower system

The absolute density of bacteria and microbiome composition observed in water samples were primarily influenced by the flow rate. For example, absolute bacterial concentrations in the showerheads with flow rate of 1.8 gpm were higher and had a more homogeneous microbiome composition (**Figure 1C**) than those in lower flow showerheads (1 gpm and 1.5 gpm). In addition, the taxonomic composition of water samples from the 1.8 gpm showerhead showed a distinct inclination towards specific bacterial taxa, such as *Sphingomonas* and *Mycobacterium*, which showed the greatest relative abundances in samples (**Figure 3**). It is possible that the higher microbial abundances were due to more water exiting the showerhead and potentially causing more sloughing than the lower flow showerheads. Although changes in hydrodynamic forces within the building plumbing system are recognized as crucial in both the formation and prevention of biofilms (Lau and Liu, 1993; Thomen et al., 2017; Tsagkari and Sloan, 2018; Cowle et al., 2020; Tsagkari et al., 2022), the impacts and relationships resulting from changes in flow rate seems unclear. For instance, a high flow rate can quickly supply nutrients and oxygen to biofilm inducing its development. At the same time, high flow rates minimize the opportunity for microorganisms to attach to surfaces due to the fast flow, deliver high concentrations of disinfectants, and induce shear stresses that inhibit the development of thick biofilms. In our study, other hydrodynamic factors related to inhibition of biofilm development other than flow rate were not explored, so future work should explore this further. Conversely, showerheads with lower flow rates contained lower concentrations of overall bacteria but had more diverse compositions (**Figures 1C**, **3**). Particularly, we observed the level of LPS biosynthesis was significantly higher in the samples with lower flow rates (**Figure 4C**), which suggests that flow rate may be selecting for microorganisms that more readily form biofilms (Lau et al., 2009). Furthermore, the relative abundance of *Methylobacterium* from the samples with lower flow rates increased after 38 days of continuous use. *Methylobacterium* is commonly found in building plumbing and participate biofilm formation (Rice et al., 2000; Feazel et al., 2009; Tsagkari et al., 2017; Gebert et al., 2018; Tsagkari and Sloan, 2018; Szwetkowski and Falkinham, 2020). Similarly, it is presumed to participate in biofilm formation and proliferated within it in the samples with lower flow rates. Interestingly, the relative abundance of *Methylobacterium* was negligible at 1.8 gpm. Nonetheless, given the observed suppression of *Methylobacterium* growth and the low LPS expression levels in the high flow samples in this study, it is hypothesized that biofilm formation was higher in the lower flow showerhead samples. Since biofilms are a known reservoir of microorganisms that are capable of causing infection (Feazel et al., 2009; Gebert et al., 2018), this increase in formation could cause biofilm sloughing after 8 weeks and contribute to greater exposure risk.

Aerosolization partitioning from low flow showerheads varied by flow rate and DWPI, with partitioning trends generally being consistent with other data taken from the INHALE shower laboratory (Pitell and Haig, 2023). These partitioning ratios suggest that even though the absolute densities of DWPIs were not significantly affected by flow rate in the aerosol samples, the likelihood of a respirable aerosol containing NTM being generated is higher in low flow showerheads and the opposite for *L. pneumophila*: respirable bioaerosol generation was highest in the highest flow showerhead. Overall, showerheads with a flow rate of 1.5 gpm seemed to have less DWPI partitioning while still reducing water use, which may be due to the optimization of lower flow rate and reduced shear stress on the biofilm forming within the fixture. While many factors such as number of water jets, orientation of jets on the showerhead, and spray pattern (Zhou et al., 2019) are known to impact aerosolization, these parameters were controlled for between the showerheads so that the effects of only flow rate were studied. Other work that has reported partitioning in this system has studied 1.8 gpm ABS plastic showerheads and found that partitioning of NTM in particular was three orders of magnitude lower in the previous study compared to the data in this paper (Pitell and Haig, 2023). The most likely explanation for these differences in aerosolization is that the INHALE shower laboratory, and possibly the biofilm growing within it, has changed over time: samples described in this study were collected in 2020, and samples in this chapter were taken in 2022. In between these two sampling endeavors, there were varying levels of use that would have aged the plumbing system between the two sampling campaigns, which may have influenced the biofilm to behave more like an established plumbing system. There may also be more NTM-specific reasons based on their growth dynamics and behavior. Despite NTM showing a strong preference for surface adhesion in pure culture within 6 hours, many NTM species have long generation times, which could cause the increase in abundance seen between these two sampling endeavors (Falkinham, 2018). Additionally, NTM is known to survive intracellularly in certain species of amoeba, so it is possible that the lower NTM partitioning in earlier sampling endeavors were caused by greater numbers of the overall NTM community proliferating in amoeba, where aerosolization would be less likely (Ovrutsky et al., 2013).

## Conclusion

DWPIs, which are prevalent in building plumbing systems and can be aerosolized through showerheads, are significantly important to manage to preserve public health. Despite this, there hasn’t been an evaluation of the health risks associated with modifications in shower systems, such as strategies for water conservation by altering the flow rate of showerheads. In this study, we analyzed the abundance and composition of bacterial communities in shower water and shower aerosols, based on the showerhead’s flow rate. We found that overall microbial densities in water increased as a function of flow rate, but abundances in aerosols were unaffected. Showerhead flow rate significantly altered the microbial communities exiting in the water and aerosol phases, with aerosols produced from the lowest flow rate showerheads (1 gpm) containing more potentially pathogenic gram-negative bacteria and higher DWPI partitioning behavior than faster flow rate showerheads. Future work should focus on assessing the pathogenicity of these aerosols to help inform microbial risk assessment tools. Based on these results, consumers may want to use showerheads with a 1.5 gpm flow rate to optimize water conservation and DWPI emission. Additionally, vulnerable populations may want to use conventional flow showerheads to minimize DWPI partitioning, and thus reduce the microbial load in aerosols. Policymakers in water-constrained areas should consider how water conserving showerheads change the water and aerosol microbiome, and use this information to wholistically convey the benefits and drawbacks of using low flow showerheads.

There are many additional avenues of investigation that must be considered in order to better understand how water conserving showerheads affect the microbiome. This work was only conducted over the course of 8 weeks, which does not encapsulate a showerhead’s operational lifetime and necessitates longer sampling periods to accurately characterize average showering dynamics. Additionally, some of the observations could be attributed to the virgin nature of the INHALE shower laboratory itself: microbial dynamics may be different in more established plumbing systems, and could explain discrepancies found in this work compared to other studies. Finally, this work showcases the need to sample respirable aerosols in addition to water for these types of studies in order to most accurately ascertain risk.

## Data availability statement

The datasets presented in this study can be found in online repositories. The names of the repository/repositories and accession number(s) can be found below: zenodo.com, doi 10.5281/zenodo.10601319.

## Author contributions

SP: Data curation, Formal analysis, Investigation, Methodology, Supervision, Visualization, Writing – original draft, Writing – review & editing. CW: Formal analysis, Visualization, Writing – original draft, Writing – review & editing. ET: Data curation, Investigation, Methodology, Writing – original draft. S-JH: Conceptualization, Funding acquisition, Project administration, Resources, Supervision, Writing – review & editing.
