# Supplementary material for: Balancing water conservation and health: do water-saving showerheads impact the microbes we breathe in during showering?
Source: Front Microbiomes. 2024 Jul 15;3:1416055. doi: 10.3389/frmbi.2024.1416055 (PMC12993522; doi:10.3389/frmbi.2024.1416055)
Supplement: Supplementary file 1 [file DataSheet_1.docx]

Supplementary Material

Balancing Water Conservation and Health: Do water-saving showerheads impact the microbes we breathe in during showering?

Cheolwoon Woo^1^, Sarah Pitell^1^, Evan Trump^1^, Sarah-Jane Haig^1,2^

^1^University of Pittsburgh, Department of Civil and Environmental Engineering, Pittsburgh, PA, USA

^2^University of Pittsburgh, School of Public Health, Pittsburgh, PA, USA

*** Correspondence:**Sarah-Jane Haig
sjhaig@pitt.edu

**Table S1.** Primer pairs, target genes, and conditions used to perform droplet digital PCR.

| Target | | Forward | Reverse | Limits of detection and quantification (copies/20 µL) | Threshold for water samples | Threshold for aerosol samples | Reference |
| --- | --- | --- | --- | --- | --- | --- | --- |
| *Legionella pneumophila* | *Lmip* gene | CCGATGCC  ACATCATAGC | CCAATTGAG  CGCCACTCATAG | 6.08 | 8800 | 6500 | (Wullings et al., 2011) |
| *Nontuberculous mycobacteria* | *atpE* gene | CCAATTGAG  CGCCACTCATAG | CGAAGACGA  ACARSGCCAT | 5.6 | 10600 | 9650 | (Radomski et al., 2013) |
| *Total bacteria* | *16s rRNA* gene | ACTCCTACG  GGAGGCAG | ATTACCGCG  GCTGCTGG | 5.3 | 12900 | 11100 | (Fierer et al., 2005) |
| Fierer, N., Jackson, J. A., Vilgalys, R., and Jackson, R. B. (2005). Assessment of Soil Microbial Community Structure by Use of Taxon-Specific Quantitative PCR Assays. *Appl. Environ. Microbiol.* 71, 4117–4120. doi: 10.1128/AEM.71.7.4117-4120.2005  Radomski, N., Roguet, A., Lucas, F. S., Veyrier, F. J., Cambau, E., Accrombessi, H., et al. (2013). atpE gene as a new useful specific molecular target to quantify Mycobacteriumin environmental samples. *BMC Microbiol.* 13, 277. doi: 10.1186/1471-2180-13-277  Wullings, B. A., Bakker, G., and Kooij, D. van der (2011). Concentration and Diversity of Uncultured *Legionella* spp. in Two Unchlorinated Drinking Water Supplies with Different Concentrations of Natural Organic Matter. *Appl. Environ. Microbiol.* 77, 634–641. doi: 10.1128/AEM.01215-10 | | | | | | | |

**Table S2.** Sample metadata and the number of bacterial sequence reads.

| Sample ID | Date (MM/DD/YY) | Flow rate (g/min) | Age | Number of sequence reads (air) | Number of sequence reads (water) |
| --- | --- | --- | --- | --- | --- |
| May-11-1a | 05/11/22 | 1.5 | 3 | 9,748 | 138,231 |
| May-11-2a | 05/11/22 | 1 | 3 | 9,684 | 160,343 |
| May-12-1b | 05/12/22 | 1 | 3 | 4,486 | 45,469 |
| May-12-1c | 05/12/22 | 1.8 | 3 | 15,936 | 106,523 |
| May-12-2b | 05/12/22 | 1.8 | 3 | 69,754 | 167,777 |
| May-12-2c | 05/12/22 | 1.5 | 3 | 12,660 | 140,213 |
| May-12-3a | 05/12/22 | 1.5 | 3 | 8,315 | 158,116 |
| May-12-3b | 05/12/22 | 1 | 3 | 74,826 | 132,804 |
| May-13-3c | 05/13/22 | 1.8 | 3 | 4,273 | 120,224 |
| May-18-1a | 05/18/22 | 1.5 | 10 | 4,142 | 21,467 |
| May-18-2a | 05/18/22 | 1 | 10 | 12,258 | 135,117 |
| May-20-1b | 05/20/22 | 1 | 10 | 39,350 | 156,307 |
| May-20-1c | 05/20/22 | 1.8 | 10 | 11,261 | 88,825 |
| May-20-2b | 05/20/22 | 1.8 | 10 | 16,259 | 274,775 |
| May-20-2c | 05/20/22 | 1.5 | 10 | 1,859 | 20,328 |
| May-20-3a | 05/20/22 | 1.5 | 10 | 2,484 | 222,144 |
| May-20-3b | 05/20/22 | 1 | 10 | 4,139 | 116,482 |
| May-21-3c | 05/21/22 | 1.8 | 10 | 7,406 | 71,274 |
| May-25-1a | 05/25/22 | 1.5 | 17 | 25,866 | 31,812 |
| May-25-2a | 05/25/22 | 1 | 17 | 40,362 | 22,449 |
| May-26-1b | 05/26/22 | 1 | 17 | 47,978 | 104,579 |
| May-26-1c | 05/26/22 | 1.8 | 17 | 55,036 | 127,223 |
| May-26-2b | 05/26/22 | 1.8 | 17 | 13,948 | 81,589 |
| May-26-2c | 05/26/22 | 1.5 | 17 | 30,436 | 164,408 |
| May-26-3a | 05/26/22 | 1.5 | 17 | 6,655 | 65,041 |
| May-26-3b | 05/26/22 | 1 | 17 | 25,527 | 55,383 |
| May-27-3c | 05/27/22 | 1.8 | 17 | 4,922 | 68,999 |
| Jun-15-1a | 06/15/22 | 1.5 | 38 | 26,011 | n.a. |
| Jun-15-2a | 06/15/22 | 1 | 38 | 27,233 | 79,490 |
| Jun-16-1b | 06/16/22 | 1 | 38 | 7,712 | n.a. |
| Jun-16-1c | 06/16/22 | 1.8 | 38 | 15,512 | n.a. |
| Jun-16-2b | 06/16/22 | 1.8 | 38 | 6,783 | 63,589 |
| Jun-16-2c | 06/16/22 | 1.5 | 38 | 32,770 | 44,513 |
| Jun-16-3a | 06/16/22 | 1.5 | 38 | 83,578 | 185,615 |
| Jun-16-3b | 06/16/22 | 1 | 38 | 4413 | 208,581 |
| Jun-17-3c | 06/17/22 | 1.8 | 38 | 4,911 | 125,030 |
| Jun-22-1a | 06/22/22 | 1.5 | 45 | 3,430 | 100,361 |
| Jun-22-2a | 06/22/22 | 1 | 45 | 4,417 | 33,248 |
| Jun-23-1b | 06/23/22 | 1 | 45 | 4,920 | 114,196 |
| Jun-23-1c | 06/23/22 | 1.8 | 45 | 44,999 | 90,678 |
| Jun-23-2b | 06/23/22 | 1.8 | 45 | 2,399 | 46,285 |
| Jun-23-2c | 06/23/22 | 1.5 | 45 | n.a. | 44,947 |
| Jun-23-3a | 06/23/22 | 1.5 | 45 | 6,202 | 92,342 |
| Jun-23-3b | 06/23/22 | 1 | 45 | 32,035 | 42,060 |
| Jun-24-3c | 06/24/22 | 1.8 | 45 | 7,524 | 36,611 |
| Jun-29-1a | 06/29/22 | 1.5 | 52 | 35,481 | 36,620 |
| Jun-29-2a | 06/29/22 | 1 | 52 | 68 | 33,536 |
| Jun-30-1b | 06/30/22 | 1 | 52 | 19,944 | 34,629 |
| Jun-30-1c | 06/30/22 | 1.8 | 52 | 39,159 | 50,540 |
| Jun-30-2b | 06/30/22 | 1.8 | 52 | 43,126 | 32,552 |
| Jun-30-2c | 06/30/22 | 1.5 | 52 | 35,398 | 71,299 |
| Jun-30-3a | 06/30/22 | 1.5 | 52 | 27,049 | 100,413 |
| Jun-30-3b | 06/30/22 | 1 | 52 | 36,562 | 87,915 |
| Jul-1-3c | 07/01/22 | 1.8 | 52 | 46,116 | 58,702 |
| Total |  |  |  | 1,157,322 | 4,811,654 |
| Average |  |  |  | 21,836 | 94,346 |
| Minimum |  |  |  | 68 | 20,328 |
| Maximum |  |  |  | 83,578 | 274,775 |

Abbreviation: n.a. not PCR-amplified.

**Table S3.** Descriptive statistics of α diversity indicators of each sample type (at the OTU level)^a^.

| Sample type | Statistics | Observed | Chao1 | ACE | Shannon | Simpson | InvSimpson | Fisher |
| --- | --- | --- | --- | --- | --- | --- | --- | --- |
| Air (n=52) | Average | 65.8 | 68.9 | 69.6 | 2.95 | 0.87 | 13.01 | 14.71 |
|  | Median | 46.0 | 48.8 | 47.2 | 2.90 | 0.90 | 10.25 | 8.60 |
|  | Minimum | 13.0 | 13.0 | 13.0 | 2.18 | 0.69 | 3.20 | 1.90 |
|  | Maximum | 235.0 | 243.6 | 242.4 | 5.03 | 0.99 | 99.19 | 72.18 |
| Water (n=51) | Average | 50.8 | 66.4 | 67.5 | 2.27 | 0.81 | 6.15 | 9.82 |
|  | Median | 49.0 | 59.5 | 60.0 | 2.32 | 0.83 | 5.95 | 9.30 |
|  | Minimum | 21.0 | 24.3 | 24.6 | 1.35 | 0.56 | 2.26 | 3.34 |
|  | Maximum | 86.0 | 142.4 | 148.6 | 3.01 | 0.91 | 11.38 | 18.81 |

^a^ The sequence reads were rarefied to 1,800 reads to calculate the α diversity indicators. One of the air samples, Jun-29-2a-Air, was excluded from calculating the statistics because of its low read.

**Table S4.** The concentration of airborne particles and each target bacteria in each sample.

| Sample ID | Particles with diameters  0.3–5 µm (Number per event) | Particles with diameters  2–5µm  (Number per event) | Concentration in air samples (copies/L) | | |  | Concentration in water samples (copies/L) | | |
| --- | --- | --- | --- | --- | --- | --- | --- | --- | --- |
|  |  |  | Total bacteria | *Legionella pneumophila* | Nontuberculous mycobacterium |  | Total bacteria | *Legionella pneumophila* | Nontuberculous mycobacterium |
| May-11-1a | 17,250,400,000 | 350,400,000 | 40 | 10 | 6 |  | 1,430,000 | 191 | 101,000 |
| May-11-2a | 41,670,000,000 | 3,510,000,000 | 60 | 5 | 8 |  | 8,640,000 | 296 | 515,000 |
| May-12-1b | 43,573,000,000 | 3,553,000,000 | 8,000 | 20 | 17 |  | 1,830,000 | 593 | 435,000 |
| May-12-1c | 19,906,300,000 | 482,300,000 | 53 | 0 | 3 |  | 1,290,000 | 151 | 661,000 |
| May-12-2b | 15,047,600,000 | 707,600,000 | 7,650 | 8 | 11 |  | 1,670,000 | 249 | 275,000 |
| May-12-2c | 9,017,400,000 | 144,500,000 | 51 | 10 | 4 |  | 2,420,000 | 470 | 163,000 |
| May-12-3a | 18,561,400,000 | 284,400,000 | 47 | 12 | 3 |  | 1,970,000 | 344 | 101,000 |
| May-12-3b | 28,335,500,000 | 378,500,000 | 9,867 | 3 | 17 |  | 1,560,000 | 493 | 108,000 |
| May-13-3c | 5,778,900,000 | 707,600,000 | 140 | 6 | 3 |  | 2,820,000 | 251 | 244,000 |
| May-18-1a | 7,122,800,000 | 196,800,000 | 43 | 0 | 2 |  | 1,800,000 | 0 | 64,300 |
| May-18-2a | 10,648,500,000 | 231,500,000 | 58 | 0 | 5 |  | 1,640,000 | 0 | 16,300 |
| May-20-1b | 8,089,900,000 | 221,900,000 | 150 | 1 | 4 |  | 2,290,000 | 0 | 266,000 |
| May-20-1c | 3,656,300,000 | 141,300,000 | 39 | 1 | 4 |  | 2,210,000 | 0 | 72,300 |
| May-20-2b | 3,971,600,000 | 127,100,000 | 27 | 0 | 1 |  | 2,530,000 | 0 | 498,000 |
| May-20-2c | 4,652,300,000 | 287,300,000 | 67 | 1 | 1 |  | 1,920,000 | 82 | 402,000 |
| May-20-3a | 8,384,500,000 | 208,500,000 | 38 | 1 | 3 |  | 2,060,000 | 0 | 854,000 |
| May-20-3b | 5,777,100,000 | 198,100,000 | 98 | 25 | 10 |  | 1,200,000 | 65 | 183,000 |
| May-21-3c | 3,595,710,000 | 93,010,000 | 61 | 77 | 8 |  | 1,930,000 | 90 | 67,200 |
| May-25-1a | 3,508,100,000 | 152,100,000 | 103 | 0 | 3 |  | 2,230,000 | 0 | 165,000 |
| May-25-2a | 4,375,000,000 | 224,000,000 | 103 | 1 | 6 |  | 786,000 | 0 | 74,100 |
| May-26-1b | 3,493,200,000 | 162,200,000 | 117 | 0 | 2 |  | 4,310,000 | 265 | 139,000 |
| May-26-1c | 3,367,900,000 | 200,900,000 | 84 | 0 | 7 |  | 2,980,000 | 746 | 67,300 |
| May-26-2b | 2,817,450,000 | 85,350,000 | 132 | 0 | 5 |  | 2,920,000 | 0 | 1,120,000 |
| May-26-2c | 3,329,700,000 | 205,700,000 | 93 | 1 | 6 |  | 1,040,000 | 0 | 169,000 |
| May-26-3a | 4,643,300,000 | 245,300,000 | 79 | 0 | 11 |  | 2,870,000 | 68 | 604,000 |
| May-26-3b | 4,375,000,000 | 199,200,000 | 91 | 0 | 9 |  | 1,400,000 | 76 | 323,000 |
| May-27-3c | 2,300,800,000 | 150,500,000 | 153 | 1 | 11 |  | 1,830,000 | 0 | 211,000 |
| Jun-15-1a | 15,950,000,000 | 199,000,000 | 6,978 | 0 | 0 |  | 0 | 0 | 0 |
| Jun-15-2a | 16,802,200,000 | 220,200,000 | 8,959 | 2 | 0 |  | 656,000 | 0 | 51,000 |
| Jun-16-1b | 20,608,600,000 | 283,300,000 | 8,307 | 0 | 1 |  | 0 | 0 | 0 |
| Jun-16-1c | 24,416,800,000 | 249,800,000 | 15,675 | 1 | 0 |  | 0 | 0 | 0 |
| Jun-16-2b | 19,392,000,000 | 1,335,000,000 | 6,642 | 0 | 0 |  | 5,780,000 | 69 | 637,000 |
| Jun-16-2c | 21,340,700,000 | 257,700,000 | 8,355 | 6 | 1 |  | 1,240,000 | 0 | 157,000 |
| Jun-16-3a | 19,231,500,000 | 183,500,000 | 7,392 | 12 | 1 |  | 3,990,000 | 0 | 1,760,000 |
| Jun-16-3b | 3,231,000,000 | 811,000,000 | 8,470 | 6 | 0 |  | 1,620,000 | 0 | 332,000 |
| Jun-17-3c | 21,290,000,000 | 672,000,000 | 12,559 | 8 | 0 |  | 13,500,000 | 0 | 369,000 |
| Jun-22-1a | 12,108,100,000 | 200,100,000 | 6,858 | 0 | 1 |  | 1,650,000 | 0 | 72,900 |
| Jun-22-2a | 12,427,000,000 | 192,000,000 | 5,397 | 0 | 3 |  | 970,000 | 70 | 48,700 |
| Jun-23-1b | 13,338,700,000 | 281,700,000 | 6,610 | 0 | 0 |  | 1,190,000 | 0 | 240,000 |
| Jun-23-1c | 16,620,200,000 | 235,200,000 | 8,669 | 2 | 3 |  | 1,900,000 | 0 | 94,100 |
| Jun-23-2b | 11,648,400,000 | 308,400,000 | 5,478 | 0 | 1 |  | 9,840,000 | 0 | 1,210,000 |
| Jun-23-2c | 14,309,700,000 | 177,700,000 | 5,652 | 0 | 0 |  | 992,000 | 0 | 108,000 |
| Jun-23-3a | 13,062,800,000 | 286,800,000 | 3,317 | 1 | 0 |  | 2,490,000 | 0 | 511,000 |
| Jun-23-3b | 12,821,700,000 | 291,700,000 | 7,164 | 0 | 2 |  | 1,070,000 | 74 | 114,000 |
| Jun-24-3c | 13,747,600,000 | 177,600,000 | 5,656 | 2 | 1 |  | 98,200,000 | 0 | 1,130,000 |
| Jun-29-1a | 10,915,300,000 | 213,300,000 | 9,808 | 0 | 4 |  | 2,080,000 | 0 | 78,300 |
| Jun-29-2a | 11,819,000,000 | 200,000,000 | 11,061 | 0 | 3 |  | 1,050,000 | 0 | 56,500 |
| Jun-30-1b | 7,554,400,000 | 241,400,000 | 6,530 | 0 | 0 |  | 766,000 | 0 | 97,800 |
| Jun-30-1c | 8,690,000,000 | 719,000,000 | 7,516 | 1 | 0 |  | 1,740,000 | 75 | 127,000 |
| Jun-30-2b | 7,270,000,000 | 169,000,000 | 9,698 | 0 | 7 |  | 3,110,000 | 0 | 1,010,000 |
| Jun-30-2c | 8,240,000,000 | 895,000,000 | 14,888 | 2 | 13 |  | 1,180,000 | 86 | 167,000 |
| Jun-30-3a | 11,866,300,000 | 221,300,000 | 9,841 | 5 | 26 |  | 3,810,000 | 146 | 479,000 |
| Jun-30-3b | 9,353,800,000 | 291,800,000 | 12,105 | 6 | 7 |  | 3,460,000 | 69 | 131,000 |
| Jul-1-3c | 8,030,000,000 | 622,000,000 | 12,632 | 7 | 12 |  | 7,570,000 | 0 | 755,000 |
| Average | 12,098,804,815 | 433,047,407 | 4,808 | 5 | 5 |  | 4,285,741 | 93 | 326,570 |
| Minimum | 2,300,800,000 | 85,350,000 | 27 | 0 | 0 |  | 0 | 0 | 0 |
| Maximum | 43,573,000,000 | 3,553,000,000 | 15,675 | 77 | 26 |  | 98,200,000 | 746 | 1,760,000 |

**Table S5.** Indicators for shower water quality.

| Sample ID | Temperature (℃) | pH | Free chlorine (mg/L) | Total chlorine (mg/L) |
| --- | --- | --- | --- | --- |
| May-11-1a | 28.1 | 7.6 | 0 | 0 |
| May-11-2a | 27.3 | 7.47 | 0.16 | 0.18 |
| May-12-1b | 30.8 | 7.38 | 0.05 | 0.09 |
| May-12-1c | 36.2 | 7.25 | 0.19 | 0.19 |
| May-12-2b | 30.2 | 7.35 | 0.15 | 0.12 |
| May-12-2c | 31 | 7.4 | 0.18 | 0.16 |
| May-12-3a | 31.8 | 7.36 | 0.05 | 0.08 |
| May-12-3b | 31.1 | 7.34 | 0.27 | 0.28 |
| May-13-3c | 31.5 | 7.43 | 0.13 | 0.18 |
| May-18-1a | 33 | 7.37 | 0.27 | 0.31 |
| May-18-2a | 32.4 | 7.15 | 0.22 | 0.27 |
| May-20-1b | 29.7 | 7.54 | 0.11 | 0.26 |
| May-20-1c | 31.5 | 7.44 | 0.31 | 0.32 |
| May-20-2b | 27.9 | 7.64 | 0.09 | 0.15 |
| May-20-2c | 28.8 | 7.54 | 0.25 | 0.24 |
| May-20-3a | 31.4 | 7.5 | 0.05 | 0.12 |
| May-20-3b | 30.2 | 7.49 | 0.24 | 0.3 |
| May-21-3c | 32.5 | 7.36 | 0.18 | 0.39 |
| May-25-1a | 31.1 | 7.02 | 0.3 | 0.38 |
| May-25-2a | 28.4 | 7.07 | 0.31 | 0.33 |
| May-26-1b | 30.5 | 6.86 | 0.23 | 0.33 |
| May-26-1c | 30 | 6.8 | 0.38 | 0.39 |
| May-26-2b | 28.7 | 6.87 | 0.19 | 0.27 |
| May-26-2c | 30.1 | 6.68 | 0.38 | 0.33 |
| May-26-3a | 29.7 | 6.75 | 0.14 | 0.19 |
| May-26-3b | 30.3 | 6.76 | 0.3 | 0.44 |
| May-27-3c | 32.8 | 6.68 | 0.21 | 0.1 |
| Jun-15-1a | 30.4 | 6.73 | 0.23 | 0.15 |
| Jun-15-2a | 30 | 6.7 | 0.36 | 0.3 |
| Jun-16-1b | 27.9 | 6.83 | 0.15 | 0.22 |
| Jun-16-1c | 29.4 | 6.85 | 0.19 | 0.27 |
| Jun-16-2b | 28 | 6.85 | 0.11 | 0.15 |
| Jun-16-2c | 28.9 | 6.87 | 0.22 | 0.31 |
| Jun-16-3a | 29 | 6.7 | 0.07 | 0.14 |
| Jun-16-3b | 30.5 | 6.83 | 0.21 | 0.08 |
| Jun-17-3c | 32 | 6.8 | 0.08 | 0 |
| Jun-22-1a | 30.2 | 6.69 | 0.22 | 0.26 |
| Jun-22-2a | 30 | 6.75 | 0.29 | 0.4 |
| Jun-23-1b | 29.9 | 6.72 | 0.19 | 0.29 |
| Jun-23-1c | 29.4 | 6.87 | 0.24 | 0.3 |
| Jun-23-2b | 29.6 | 6.87 | 0.27 | 0.28 |
| Jun-23-2c | 28.7 | 6.82 | 0.12 | 0.1 |
| Jun-23-3a | 30.7 | 6.75 | 0.1 | 0.14 |
| Jun-23-3b | 31 | 6.77 | 0.32 | 0.35 |
| Jun-24-3c | 31.3 | 6.79 | 0.16 | 0.2 |
| Jun-29-1a | 30.3 | 7.52 | 0.36 | 0.39 |
| Jun-29-2a | 30.3 | 6.6 | 0.3 | 0.45 |
| Jun-30-1b | 28.6 | 7.28 | 0.18 | 0.27 |
| Jun-30-1c | 30.1 | 7.61 | 0.3 | 0.4 |
| Jun-30-2b | 30.1 | 7.31 | 0.22 | 0.24 |
| Jun-30-2c | 29.6 | 7.34 | 0.32 | 0.39 |
| Jun-30-3a | 29.6 | 6.95 | 0.19 | 0.27 |
| Jun-30-3b | 30.5 | 7.35 | 0.34 | 0.41 |
| Jul-1-3c | 32.3 | 6.77 | 0.17 | 0.29 |
| Average | 30.3 | 7.07 | 0.21 | 0.25 |
| Minimum | 27.3 | 6.6 | 0 | 0 |
| Maximum | 36.2 | 7.64 | 0.38 | 0.45 |

**Table S6.** Metals in shower water. All values are in mg/L.

| Sample ID | Total | | | | | |  | Dissolved | | | | | |
| --- | --- | --- | --- | --- | --- | --- | --- | --- | --- | --- | --- | --- | --- |
|  | Magnesium | Calcium | Iron | Copper | Silver | Lead |  | Magnesium | Calcium | Iron | Copper | Silver | Lead |
| May-11-1a | 6.22 | 27.50 | 0.18 | 0.05 | 0.02 | 3.7 × 10^-3^ |  | 6.20 | 28.41 | 0.16 | 0.03 | n.d. | 3.0 × 10^-4^ |
| May-11-2a | 5.99 | 25.24 | 0.14 | 0.03 | n.d. | 3.7 × 10^-3^ |  | 6.40 | 26.85 | 0.15 | 0.04 | 0.01 | 3.3 × 10^-4^ |
| May-12-1b | 5.27 | 24.11 | 0.13 | 0.03 | n.d. | 3.5 × 10^-3^ |  | 5.77 | 26.63 | 0.14 | 0.02 | n.d. | 2.7 × 10^-4^ |
| May-12-1c | 5.58 | 26.15 | 0.14 | 0.03 | n.d. | 5.8 × 10^-4^ |  | 5.16 | 24.44 | 0.12 | 0.02 | n.d. | 2.1 × 10^-4^ |
| May-12-2b | 5.75 | 25.98 | 0.14 | 0.04 | n.d. | 4.3 × 10^-4^ |  | 6.07 | 26.95 | 0.15 | 0.03 | n.d. | 3.6 × 10^-4^ |
| May-12-2c | 5.16 | 25.11 | 0.14 | 0.08 | n.d. | 3.4 × 10^-4^ |  | 4.72 | 23.21 | 0.11 | 0.05 | n.d. | 3.1 × 10^-4^ |
| May-12-3a | 6.45 | 28.65 | 0.17 | 0.04 | n.d. | 3.4 × 10^-4^ |  | 5.75 | 25.91 | 0.13 | 0.02 | 5.6 × 10^-3^ | 2.2 × 10^-4^ |
| May-12-3b | 5.79 | 28.12 | 0.16 | 0.05 | n.d. | 3.0 × 10^-4^ |  | 5.22 | 25.37 | 0.14 | 0.03 | 8.6 × 10^-4^ | 2.0 × 10^-4^ |
| May-13-3c | 6.22 | 33.83 | 0.19 | 0.04 | n.d. | 3.8 × 10^-4^ |  | 5.42 | 25.96 | 0.13 | 0.03 | n.d. | 2.2 × 10^-4^ |
| May-18-1a | 3.92 | 22.38 | 0.14 | 0.03 | n.d. | 4.7 × 10^-4^ |  | 5.72 | 27.10 | 0.15 | 0.03 | n.d. | 2.6 × 10^-4^ |
| May-18-2a | 5.49 | 27.38 | 0.16 | 0.05 | 3.2 × 10^-4^ | 7.9 × 10^-4^ |  | 5.42 | 26.24 | 0.14 | 0.03 | n.d. | 2.7 × 10^-4^ |
| May-20-1b | 6.03 | 26.36 | 0.15 | 0.04 | n.d. | 3.8 × 10^-4^ |  | 5.96 | 27.52 | 0.14 | 0.03 | n.d. | 6.8 × 10^-4^ |
| May-20-1c | 5.72 | 27.23 | 0.14 | 0.04 | n.d. | 2.6 × 10^-4^ |  | 5.59 | 27.27 | 0.14 | 0.03 | n.d. | 2.4 × 10^-4^ |
| May-20-2b | 6.27 | 27.34 | 0.16 | 0.03 | n.d. | 4.4 × 10^-4^ |  | 6.04 | 27.30 | 0.14 | 0.02 | n.d. | 2.6 × 10^-4^ |
| May-20-2c | 5.94 | 28.03 | 0.16 | 0.05 | n.d. | 3.2 × 10^-4^ |  | 5.88 | 27.47 | 0.15 | 0.04 | n.d. | 6.8 × 10^-3^ |
| May-20-3a | 6.25 | 27.48 | 0.15 | 0.04 | n.d. | 5.0 × 10^-4^ |  | 5.79 | 25.79 | 0.13 | 0.02 | n.d. | 8.7 × 10^-3^ |
| May-20-3b | 5.99 | 27.80 | 0.15 | 0.05 | n.d. | 4.0 × 10^-4^ |  | 5.73 | 26.66 | 0.14 | 0.03 | n.d. | 3.1 × 10^-4^ |
| May-21-3c | 6.09 | 27.59 | 0.15 | 0.04 | n.d. | 3.3 × 10^-4^ |  | 5.88 | 26.94 | 0.15 | 0.03 | n.d. | 2.4 × 10^-4^ |
| May-25-1a | 6.96 | 29.96 | 0.17 | 0.05 | n.d. | 3.4 × 10^-4^ |  | 6.82 | 29.15 | 0.16 | 0.04 | n.d. | 5.1 × 10^-4^ |
| May-25-2a | 6.85 | 29.85 | 0.18 | 0.05 | n.d. | 6.1 × 10^-3^ |  | 6.74 | 29.78 | 0.15 | 0.03 | n.d. | 1.9 × 10^-3^ |
| May-26-1b | 7.14 | 30.21 | 0.17 | 0.04 | n.d. | 4.3 × 10^-4^ |  | 6.95 | 29.52 | 0.17 | 0.03 | n.d. | 4.6 × 10^-4^ |
| May-26-1c | 7.33 | 30.51 | 0.18 | 0.04 | n.d. | 4.8 × 10^-4^ |  | 7.25 | 30.39 | 0.19 | 0.04 | n.d. | 6.6 × 10^-4^ |
| May-26-2b | 7.75 | 32.94 | 0.19 | 0.05 | n.d. | 5.8 × 10^-4^ |  | 7.18 | 28.99 | 0.16 | 0.03 | n.d. | 5.8 × 10^-4^ |
| May-26-2c | 7.16 | 29.22 | 0.18 | 0.04 | n.d. | 5.2 × 10^-4^ |  | 6.90 | 30.71 | 0.16 | 0.03 | n.d. | 1.0 × 10^-3^ |
| May-26-3a | 7.23 | 28.95 | 0.17 | 0.04 | n.d. | 0.04 |  | 7.18 | 29.29 | 0.16 | 0.03 | n.d. | 2.8 × 10^-4^ |
| May-26-3b | 7.09 | 30.29 | 0.17 | 0.04 | n.d. | 0.09 |  | 7.12 | 29.97 | 0.16 | 0.03 | n.d. | 3.1 × 10^-4^ |
| May-27-3c | 7.32 | 30.23 | 0.17 | 0.04 | n.d. | 0.03 |  | 7.60 | 31.51 | 0.18 | 0.12 | n.d. | 1.9 × 10^-3^ |
| Jun-15-1a | 9.31 | 71.07 | 0.42 | 0.06 | n.d. | 4.0 × 10^-3^ |  | 7.86 | 38.05 | 0.19 | 0.05 | n.d. | 6.9 × 10^-4^ |
| Jun-15-2a | 8.26 | 33.84 | 0.19 | 0.07 | n.d. | 4.3 × 10^-3^ |  | 7.39 | 31.81 | 0.19 | 0.05 | n.d. | 3.2 × 10^-4^ |
| Jun-16-1b | 7.80 | 33.93 | 0.22 | 0.07 | n.d. | 6.3 × 10^-4^ |  | 7.52 | 32.89 | 0.19 | 0.06 | n.d. | 1.3 × 10^-3^ |
| Jun-16-1c | 7.67 | 33.55 | 0.20 | 0.07 | n.d. | 3.9 × 10^-4^ |  | 7.59 | 33.52 | 0.18 | 0.05 | n.d. | 0.23 |
| Jun-16-2b | 7.80 | 34.31 | 0.20 | 0.06 | n.d. | 0.15 |  | 7.09 | 30.29 | 0.17 | 0.04 | n.d. | 0.09 |
| Jun-16-2c | 7.81 | 33.66 | 0.19 | 0.07 | n.d. | 3.3 × 10^-4^ |  | 7.79 | 32.68 | 0.22 | 0.06 | n.d. | 1.5 × 10^-3^ |
| Jun-16-3a | 7.64 | 32.44 | 0.19 | 0.05 | n.d. | 5.9 × 10^-4^ |  | 7.76 | 33.17 | 0.18 | 0.05 | n.d. | 4.6 × 10^-3^ |
| Jun-16-3b | 7.93 | 33.70 | 1.01 | 0.07 | n.d. | 5.9 × 10^-3^ |  | 8.08 | 39.27 | 0.19 | 0.05 | n.d. | 6.8 × 10^-4^ |
| Jun-17-3c | 7.89 | 32.74 | 0.21 | 0.05 | n.d. | 4.7 × 10^-4^ |  | 7.81 | 33.26 | 0.19 | 0.07 | n.d. | 2.8 × 10^-3^ |
| Jun-22-1a | 7.79 | 32.16 | 0.21 | 0.04 | n.d. | 5.1 × 10^-4^ |  | 7.16 | 31.30 | 0.17 | 0.04 | n.d. | 0.01 |
| Jun-22-2a | 7.78 | 33.93 | 0.23 | 0.05 | n.d. | 7.6 × 10^-4^ |  | 7.69 | 33.17 | 0.20 | 0.04 | n.d. | 4.3 × 10^-4^ |
| Jun-23-1b | 7.89 | 33.81 | 0.22 | 0.05 | n.d. | 8.9 × 10^-4^ |  | 7.67 | 31.26 | 0.17 | 0.04 | n.d. | 5.2 × 10^-4^ |
| Jun-23-1c | 7.59 | 32.45 | 0.19 | 0.07 | n.d. | 5.1 × 10^-4^ |  | 7.69 | 33.76 | 0.20 | 0.06 | 5.8 × 10^-4^ | 1.0 × 10^-3^ |
| Jun-23-2b | 7.28 | 32.30 | 0.18 | 0.07 | n.d. | 5.1 × 10^-3^ |  | 7.49 | 32.74 | 0.19 | 0.06 | n.d. | 4.2 × 10^-4^ |
| Jun-23-2c | 7.60 | 33.14 | 0.20 | 0.05 | n.d. | 2.3 × 10^-3^ |  | 7.58 | 32.12 | 0.18 | 0.04 | n.d. | 2.9 × 10^-4^ |
| Jun-23-3a | 7.85 | 32.53 | 0.19 | 0.04 | n.d. | 3.9 × 10^-4^ |  | 8.22 | 34.14 | 0.18 | 0.03 | n.d. | 3.5 × 10^-4^ |
| Jun-23-3b | 7.75 | 33.58 | 0.33 | 0.06 | n.d. | 4.5 × 10^-4^ |  | 7.16 | 31.32 | 0.22 | 0.04 | n.d. | 6.6 × 10^-4^ |
| Jun-24-3c | 7.86 | 33.69 | 0.36 | 0.05 | n.d. | 1.7 × 10^-3^ |  | 7.81 | 32.99 | 0.18 | 0.04 | n.d. | 2.1 × 10^-4^ |
| Jun-29-1a | 7.65 | 31.94 | 0.20 | 0.05 | 1.7 × 10^-3^ | 3.1 × 10^-4^ |  | 7.53 | 31.88 | 0.19 | 0.04 | n.d. | 4.6 × 10^-4^ |
| Jun-29-2a | 7.79 | 32.84 | 0.28 | 0.05 | n.d. | 7.9 × 10^-4^ |  | 7.58 | 31.87 | 0.19 | 0.04 | n.d. | 5.3 × 10^-4^ |
| Jun-30-1b | 7.96 | 32.81 | 0.21 | 0.08 | n.d. | 1.3 × 10^-3^ |  | 7.62 | 31.89 | 0.19 | 0.06 | n.d. | 3.3 × 10^-4^ |
| Jun-30-1c | 7.72 | 32.33 | 0.26 | 0.08 | n.d. | 4.5 × 10^-4^ |  | 7.66 | 31.16 | 0.19 | 0.05 | n.d. | 6.3 × 10^-4^ |
| Jun-30-2b | 7.43 | 31.09 | 0.20 | 0.06 | n.d. | 7.4 × 10^-4^ |  | 7.47 | 31.17 | 0.21 | 0.05 | n.d. | 4.1 × 10^-4^ |
| Jun-30-2c | 7.92 | 32.47 | 0.21 | 0.06 | n.d. | 3.7 × 10^-4^ |  | 7.77 | 32.15 | 0.19 | 0.05 | n.d. | 1.3 × 10^-3^ |
| Jun-30-3a | n.a. | n.a. | n.a. | n.a. | n.a. | n.a. |  | 8.03 | 31.47 | 0.28 | 0.06 | n.d. | 4.8 × 10^-4^ |
| Jun-30-3b | 8.03 | 32.35 | 0.22 | 0.06 | n.d. | 4.9 × 10^-4^ |  | 7.53 | 31.12 | 0.19 | 0.05 | n.d. | 7.1 × 10^-4^ |
| Jul-1-3c | 7.86 | 31.67 | 0.22 | 0.05 | n.d. | 1.9 × 10^-4^ |  | 7.86 | 31.57 | 0.19 | 0.04 | n.d. | 2.6 × 10^-4^ |
| Average | 7.03 | 31.18 | 0.21 | 0.05 | 3.5 × 10^-4^ | 6.8 × 10^-3^ |  | 6.91 | 30.14 | 0.17 | 0.04 | 3.5 × 10^-4^ | 6.9 × 10^-3^ |
| Minimum | 3.9 | 22.38 | 0.13 | 0.03 | n.d. | 1.9 × 10^-4^ |  | 4.72 | 23.21 | 0.11 | 0.02 | n.d. | 1.9 × 10^-4^ |
| Maximum | 9.3 | 71.07 | 1.01 | 0.08 | 0.02 | 0.15 |  | 8.22 | 39.27 | 0.28 | 0.12 | 0.01 | 0.23 |

Abbreviation: n.d. not detected, n.a. not recovered for analysis

**Table S7.** Metabolic functional features with statistically significant differences in all samples by sample type. Differential abundance analysis, using a p-value threshold of less than 0.05, compared the relative abundance of metabolic functional features between two sample types, air and water.

| Pathway | | Air (n=53) | | | Water (n=51) | | |
| --- | --- | --- | --- | --- | --- | --- | --- |
| Class (n = 10) | Name (n = 78) | Min (%) | Average (%) | Max (%) | Min (%) | Average (%) | Max (%) |
| Amino acid metabolism | Alanine, aspartate and glutamate metabolism | 1.279 | 1.416 | 1.726 | 1.155 | 1.297 | 1.407 |
|  | Valine, leucine and isoleucine degradation | 0.834 | 1.38 | 1.703 | 1.725 | 1.944 | 2.191 |
|  | Lysine degradation | 0.394 | 0.649 | 0.791 | 0.697 | 0.879 | 1.086 |
|  | Histidine metabolism | 0.636 | 0.893 | 0.969 | 0.717 | 0.772 | 0.863 |
|  | Tyrosine metabolism | 0.463 | 0.773 | 0.981 | 0.601 | 0.673 | 0.817 |
|  | Tryptophan metabolism | 0.545 | 1 | 1.274 | 1.09 | 1.238 | 1.445 |
|  |  |  |  |  |  |  |  |
| Biosynthesis of other secondary metabolites | Caffeine metabolism | 0 | 0.002 | 0.011 | 0 | 0.009 | 0.024 |
|  | Penicillin and cephalosporin biosynthesis | 0.025 | 0.055 | 0.073 | 0.002 | 0.018 | 0.033 |
|  | Novobiocin biosynthesis | 0.178 | 0.221 | 0.253 | 0.172 | 0.192 | 0.222 |
|  | Streptomycin biosynthesis | 0.360 | 0.445 | 0.608 | 0.333 | 0.413 | 0.457 |
|  | Neomycin, kanamycin and gentamicin biosynthesis | 0.056 | 0.077 | 0.166 | 0.029 | 0.05 | 0.06 |
|  | Indole alkaloid biosynthesis | 0 | 0.003 | 0.019 | 0 | 0.001 | 0.003 |
|  | Phenylpropanoid biosynthesis | 0.069 | 0.119 | 0.303 | 0.107 | 0.178 | 0.304 |
|  | Isoflavonoid biosynthesis | 0 | 0.001 | 0.007 | 0 | 0.0003 | 0.006 |
|  | Flavone and flavonol biosynthesis | 0 | 0.004 | 0.023 | 0 | 0.0003 | 0.002 |
|  | Stilbenoid, diarylheptanoid and gingerol biosynthesis | 0.004 | 0.035 | 0.057 | 0.026 | 0.09 | 0.18 |
|  | Tropane, piperidine and pyridine alkaloid biosynthesis | 0.161 | 0.245 | 0.277 | 0.172 | 0.214 | 0.245 |
|  | Betalain biosynthesis | 0.001 | 0.01 | 0.032 | 0.004 | 0.014 | 0.035 |
|  |  |  |  |  |  |  |  |
| Carbohydrate metabolism | Citrate cycle (TCA cycle) | 0.963 | 1.145 | 1.413 | 1.212 | 1.336 | 1.422 |
|  | Pentose phosphate pathway | 0.949 | 1.147 | 1.384 | 0.867 | 1.008 | 1.122 |
|  | Pentose and glucuronate interconversions | 0.457 | 0.69 | 0.827 | 0.427 | 0.546 | 0.659 |
|  | Fructose and mannose metabolism | 0.453 | 0.706 | 1.206 | 0.487 | 0.571 | 0.631 |
|  | Ascorbate and aldarate metabolism | 0.198 | 0.412 | 0.608 | 0.178 | 0.267 | 0.405 |
|  | Inositol phosphate metabolism | 0.229 | 0.408 | 0.562 | 0.259 | 0.31 | 0.38 |
|  | Propanoate metabolism | 1.097 | 1.426 | 1.602 | 1.423 | 1.699 | 2.003 |
|  | Butanoate metabolism | 1.189 | 1.504 | 1.686 | 1.679 | 1.858 | 2.033 |
|  |  |  |  |  |  |  |  |
| Energy metabolism | Photosynthesis - antenna proteins | 0 | 0.001 | 0.019 | 0 | 0.0001 | 0.003 |
|  | Methane metabolism | 1.221 | 1.470 | 1.638 | 1.077 | 1.23 | 1.413 |
|  | Carbon fixation in photosynthetic organisms | 0.712 | 0.804 | 1 | 0.648 | 0.722 | 0.813 |
|  | Carbon fixation pathways in prokaryotes | 1.344 | 1.489 | 1.844 | 1.609 | 1.735 | 1.844 |
|  | Nitrogen metabolism | 1.098 | 1.296 | 1.564 | 0.803 | 1.043 | 1.296 |
|  | Sulfur metabolism | 0.491 | 0.543 | 0.679 | 0.569 | 0.653 | 0.735 |
|  |  |  |  |  |  |  |  |
| Glycan biosynthesis and metabolism | N-Glycan biosynthesis | 0.006 | 0.029 | 0.074 | 0.034 | 0.064 | 0.097 |
|  | Various types of N-glycan biosynthesis | 0 | 0.001 | 0.004 | 0 | 0.00003 | 0.0003 |
|  | Other types of O-glycan biosynthesis | 0 | 0 | 0 | 0 | 0.00000003 | 0.000002 |
|  | Glycosaminoglycan degradation | 0.005 | 0.048 | 0.15 | 0.005 | 0.02 | 0.047 |
|  | Lipopolysaccharide biosynthesis | 0.328 | 0.607 | 0.748 | 0.065 | 0.202 | 0.378 |
|  | Glycosphingolipid biosynthesis - globo and isoglobo series | 0.007 | 0.058 | 0.172 | 0.008 | 0.024 | 0.048 |
|  | Glycosphingolipid biosynthesis - ganglio series | 0.003 | 0.032 | 0.101 | 0.002 | 0.009 | 0.024 |
|  |  |  |  |  |  |  |  |
| Lipid metabolism | Fatty acid elongation | 0 | 0.0002 | 0.002 | 0 | 0.00001 | 0.0001 |
|  | Fatty acid degradation | 0.614 | 1.096 | 1.356 | 1.193 | 1.494 | 1.886 |
|  | Primary bile acid biosynthesis | 0.01 | 0.041 | 0.066 | 0.027 | 0.059 | 0.092 |
|  | Secondary bile acid biosynthesis | 0.005 | 0.034 | 0.06 | 0.004 | 0.017 | 0.037 |
|  | Steroid hormone biosynthesis | 0.009 | 0.071 | 0.136 | 0.049 | 0.125 | 0.205 |
|  | Glycerolipid metabolism | 0.437 | 0.543 | 0.76 | 0.378 | 0.474 | 0.598 |
|  | Glycerophospholipid metabolism | 0.815 | 0.904 | 0.991 | 0.708 | 0.763 | 0.829 |
|  | Ether lipid metabolism | 0.011 | 0.09 | 0.167 | 0.005 | 0.025 | 0.072 |
|  | Arachidonic acid metabolism | 0.074 | 0.172 | 0.21 | 0.127 | 0.153 | 0.181 |
|  | Linoleic acid metabolism | 0.03 | 0.052 | 0.123 | 0.043 | 0.081 | 0.109 |
|  | alpha-Linolenic acid metabolism | 0.022 | 0.06 | 0.085 | 0.036 | 0.079 | 0.109 |
|  | Biosynthesis of unsaturated fatty acids | 0.285 | 0.409 | 0.524 | 0.451 | 0.529 | 0.612 |
|  |  |  |  |  |  |  |  |
| Metabolism of cofactors and vitamins | Ubiquinone and other terpenoid-quinone biosynthesis | 0.387 | 0.507 | 0.571 | 0.363 | 0.396 | 0.43 |
|  | Biotin metabolism | 0.167 | 0.203 | 0.292 | 0.139 | 0.241 | 0.315 |
|  | Retinol metabolism | 0.062 | 0.126 | 0.162 | 0.137 | 0.191 | 0.299 |
|  | Porphyrin metabolism | 1.026 | 1.311 | 1.54 | 1.271 | 1.575 | 1.91 |
|  |  |  |  |  |  |  |  |
| Metabolism of other amino acids | beta-Alanine metabolism | 0.352 | 0.609 | 0.77 | 0.495 | 0.732 | 1.014 |
|  | Taurine and hypotaurine metabolism | 0.208 | 0.286 | 0.36 | 0.199 | 0.242 | 0.285 |
|  | Phosphonate and phosphinate metabolism | 0.09 | 0.164 | 0.254 | 0.047 | 0.086 | 0.123 |
|  |  |  |  |  |  |  |  |
| Metabolism of terpenoids and polyketides | Biosynthesis of 12-, 14- and 16-membered macrolides | 0 | 0.0001 | 0.002 | 0 | 0.000001 | 0.00004 |
|  | Polyketide sugar unit biosynthesis | 0.139 | 0.225 | 0.295 | 0.127 | 0.173 | 0.216 |
|  | Terpenoid backbone biosynthesis | 0.635 | 0.737 | 0.917 | 0.78 | 0.875 | 0.965 |
|  | Monoterpenoid biosynthesis | 0 | 0.0001 | 0.002 | 0.003 | 0.013 | 0.028 |
|  | Limonene degradation | 0.146 | 0.357 | 0.52 | 0.351 | 0.551 | 0.832 |
|  | Brassinosteroid biosynthesis | 0 | 0.0001 | 0.003 | 0 | 0.000002 | 0.0001 |
|  | Carotenoid biosynthesis | 0.016 | 0.067 | 0.161 | 0.106 | 0.18 | 0.282 |
|  | Sesquiterpenoid and triterpenoid biosynthesis | 0 | 0.0002 | 0.002 | 0 | 0.00001 | 0.0001 |
|  | Biosynthesis of ansamycins | 0.066 | 0.148 | 0.204 | 0.054 | 0.069 | 0.115 |
|  | Biosynthesis of siderophore group nonribosomal peptides | 0.003 | 0.038 | 0.118 | 0.066 | 0.139 | 0.264 |
|  | Biosynthesis of type II polyketide backbone | 0 | 0.001 | 0.008 | 0 | 0.00001 | 0.0003 |
|  | Biosynthesis of type II polyketide products | 0 | 0.0003 | 0.003 | 0 | 0.00001 | 0.0002 |
|  |  |  |  |  |  |  |  |
| Xenobiotics biodegradation and metabolism | Polycyclic aromatic hydrocarbon degradation | 0.017 | 0.097 | 0.147 | 0.06 | 0.147 | 0.257 |
|  | Aminobenzoate degradation | 0.336 | 0.64 | 0.812 | 0.657 | 0.796 | 1.019 |
|  | Dioxin degradation | 0.038 | 0.129 | 0.2 | 0.057 | 0.104 | 0.138 |
|  | Xylene degradation | 0.03 | 0.102 | 0.167 | 0.023 | 0.072 | 0.118 |
|  | Bisphenol degradation | 0.038 | 0.136 | 0.181 | 0.135 | 0.239 | 0.35 |
|  | Metabolism of xenobiotics by cytochrome P450 | 0.154 | 0.387 | 0.475 | 0.391 | 0.488 | 0.561 |
|  | Drug metabolism - cytochrome P450 | 0.156 | 0.384 | 0.48 | 0.433 | 0.53 | 0.609 |
|  | Caprolactam degradation | 0.058 | 0.166 | 0.248 | 0.267 | 0.386 | 0.502 |

**Table S8.** Metabolic functional features with statistically significant differences in air samples by age factor. Differential abundance and *post hoc* analysis, using a p-value threshold of less than 0.05, compared the relative abundance of metabolic functional features between two groups, 3 days and 52 days.

| Pathway | | 3 days (n=9, air samples only) | | | 52 days (n=9, air samples only) | | |
| --- | --- | --- | --- | --- | --- | --- | --- |
| Class (n = 8) | Name (n = 35) | Min (%) | Average (%) | Max (%) | Min (%) | Average (%) | Max (%) |
| Amino acid metabolism | Lysine degradation | 0.394 | 0.555 | 0.686 | 0.746 | 0.765 | 0.788 |
|  | Phenylalanine metabolism | 0.426 | 0.648 | 0.85 | 0.873 | 0.904 | 0.916 |
|  | Tryptophan metabolism | 0.545 | 0.804 | 0.994 | 1.199 | 1.229 | 1.274 |
|  | Tyrosine metabolism | 0.463 | 0.64 | 0.823 | 0.905 | 0.934 | 0.981 |
|  |  |  |  |  |  |  |  |
| Biosynthesis of other secondary metabolites | Flavone and flavonol biosynthesis | 0.00003 | 0.00005 | 0.0001 | 0 | 0.00001 | 0.00002 |
|  | Flavonoid biosynthesis | 0.00002 | 0.0001 | 0.0002 | 0.0002 | 0.0003 | 0.0003 |
|  | Stilbenoid, diarylheptanoid and gingerol biosynthesis | 0.0001 | 0.0002 | 0.0003 | 0.0005 | 0.001 | 0.001 |
|  |  |  |  |  |  |  |  |
| Carbohydrate metabolism | Ascorbate and aldarate metabolism | 0.205 | 0.29 | 0.389 | 0.516 | 0.551 | 0.608 |
|  | Inositol phosphate metabolism | 0.249 | 0.315 | 0.363 | 0.49 | 0.526 | 0.562 |
|  |  |  |  |  |  |  |  |
| Glycan biosynthesis and metabolism | Glycosaminoglycan degradation | 0.022 | 0.078 | 0.146 | 0.005 | 0.008 | 0.011 |
|  | Glycosphingolipid biosynthesis - ganglio series | 0.015 | 0.052 | 0.09 | 0.003 | 0.006 | 0.008 |
|  | Glycosphingolipid biosynthesis - globo and isoglobo series | 0.034 | 0.097 | 0.166 | 0.007 | 0.011 | 0.018 |
|  | N-Glycan biosynthesis | 0.019 | 0.035 | 0.058 | 0.006 | 0.011 | 0.015 |
|  | Other glycan degradation | 0.058 | 0.208 | 0.35 | 0.034 | 0.045 | 0.057 |
|  |  |  |  |  |  |  |  |
| Lipid metabolism | Ether lipid metabolism | 0.014 | 0.041 | 0.067 | 0.121 | 0.141 | 0.167 |
|  | Fatty acid degradation | 0.614 | 0.932 | 1.15 | 1.307 | 1.328 | 1.356 |
|  | Primary bile acid biosynthesis | 0.018 | 0.033 | 0.066 | 0.045 | 0.049 | 0.058 |
|  | Secondary bile acid biosynthesis | 0.005 | 0.023 | 0.06 | 0.04 | 0.045 | 0.056 |
|  | Steroid biosynthesis | 0.015 | 0.026 | 0.034 | 0.068 | 0.078 | 0.087 |
|  | Steroid hormone biosynthesis | 0.012 | 0.027 | 0.042 | 0.102 | 0.114 | 0.136 |
|  |  |  |  |  |  |  |  |
| Metabolism of other amino acids | beta-Alanine metabolism | 0.379 | 0.516 | 0.62 | 0.728 | 0.747 | 0.77 |
|  | Phosphonate and phosphinate metabolism | 0.001 | 0.001 | 0.001 | 0.002 | 0.002 | 0.003 |
|  |  |  |  |  |  |  |  |
| Metabolism of terpenoids and polyketides | Biosynthesis of ansamycins | 0.08 | 0.109 | 0.161 | 0.165 | 0.178 | 0.204 |
|  | Biosynthesis of siderophore group nonribosomal peptides | 0.026 | 0.056 | 0.112 | 0.003 | 0.013 | 0.021 |
|  | Limonene degradation | 0.171 | 0.252 | 0.323 | 0.465 | 0.479 | 0.52 |
|  |  |  |  |  |  |  |  |
| Xenobiotics biodegradation and metabolism | Aminobenzoate degradation | 0.343 | 0.526 | 0.746 | 0.779 | 0.796 | 0.812 |
|  | Benzoate degradation | 0.475 | 0.794 | 1.102 | 1.34 | 1.383 | 1.466 |
|  | Bisphenol degradation | 0.072 | 0.112 | 0.16 | 0.168 | 0.173 | 0.18 |
|  | Chloroalkane and chloroalkene degradation | 0.268 | 0.367 | 0.451 | 0.599 | 0.619 | 0.665 |
|  | Chlorocyclohexane and chlorobenzene degradation | 0.059 | 0.149 | 0.234 | 0.315 | 0.336 | 0.368 |
|  | Dioxin degradation | 0.064 | 0.088 | 0.104 | 0.162 | 0.178 | 0.2 |
|  | Ethylbenzene degradation | 0.04 | 0.086 | 0.107 | 0.17 | 0.181 | 0.2 |
|  | Naphthalene degradation | 0.169 | 0.277 | 0.363 | 0.409 | 0.418 | 0.432 |
|  | Styrene degradation | 0.077 | 0.194 | 0.293 | 0.327 | 0.342 | 0.353 |
|  | Xylene degradation | 0.03 | 0.056 | 0.076 | 0.135 | 0.15 | 0.167 |

**Table S9.** Metabolic functional features with statistically significant differences in water samples by flow rate. Differential abundance and *post hoc* analysis, using a p-value threshold of less than 0.05, compared the relative abundance of metabolic functional features between two groups, 1 g/min and 1.8 g/min.

| Pathway | | 1 g/min  (n=9, water samples only) | | | 1.8 g/min  (n=9, water samples only) | | |
| --- | --- | --- | --- | --- | --- | --- | --- |
| Class (n = 2) | Name (n = 3) | Min (%) | Average (%) | Max (%) | Min (%) | Average (%) | Max (%) |
| Biosynthesis of other secondary metabolites | Caffeine metabolism | 0.005 | 0.011 | 0.02 | 0.0004 | 0.007 | 0.024 |
|  | Penicillin and cephalosporin biosynthesis | 0.012 | 0.021 | 0.03 | 0.002 | 0.012 | 0.027 |
|  |  |  |  |  |  |  |  |
| Glycan biosynthesis and metabolism | Lipopolysaccharide biosynthesis | 0.152 | 0.236 | 0.321 | 0.065 | 0.134 | 0.235 |


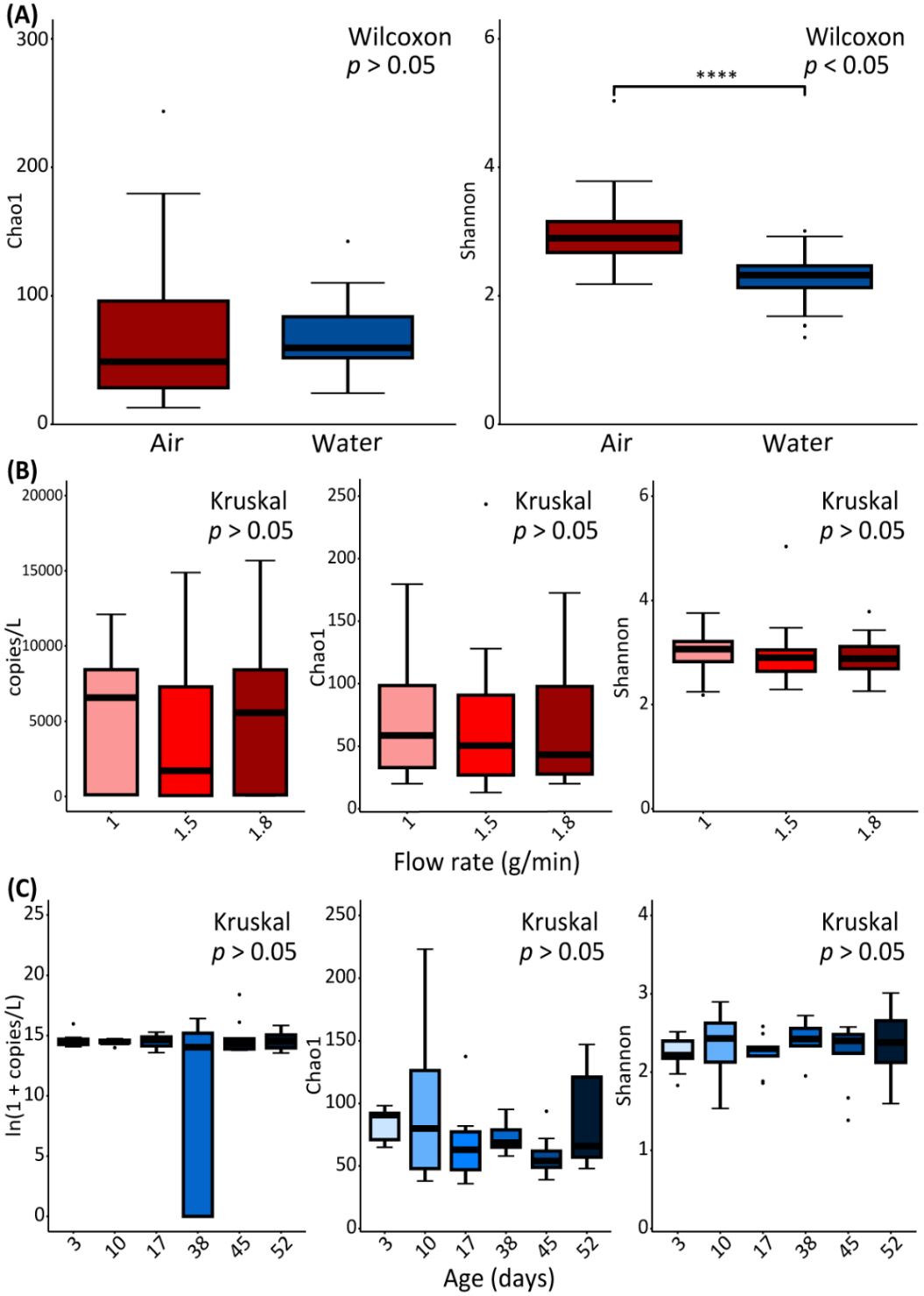


**Figure S1.** Diversity of identified bacteria. The results shown are based on the operational taxonomic units (OTUs). (A) The comparison of richness and diversity of bacterial OTUs in air and water samples estimated by the Chao1 estimator and Shannon index, respectively. (B) The concentration of bacteria in air samples by flow rate and the comparison of richness and diversity of bacterial OTUs in air samples by flow rate by the Chao1 estimator and Shannon index, respectively. (C) The concentration of bacteria in water samples over time and the comparison of richness and diversity of bacterial OTUs in water samples over time estimated by the Chao1 estimator and Shannon index, respectively. In panel (A), four asterisks (****) represent *p* < 0.0001.


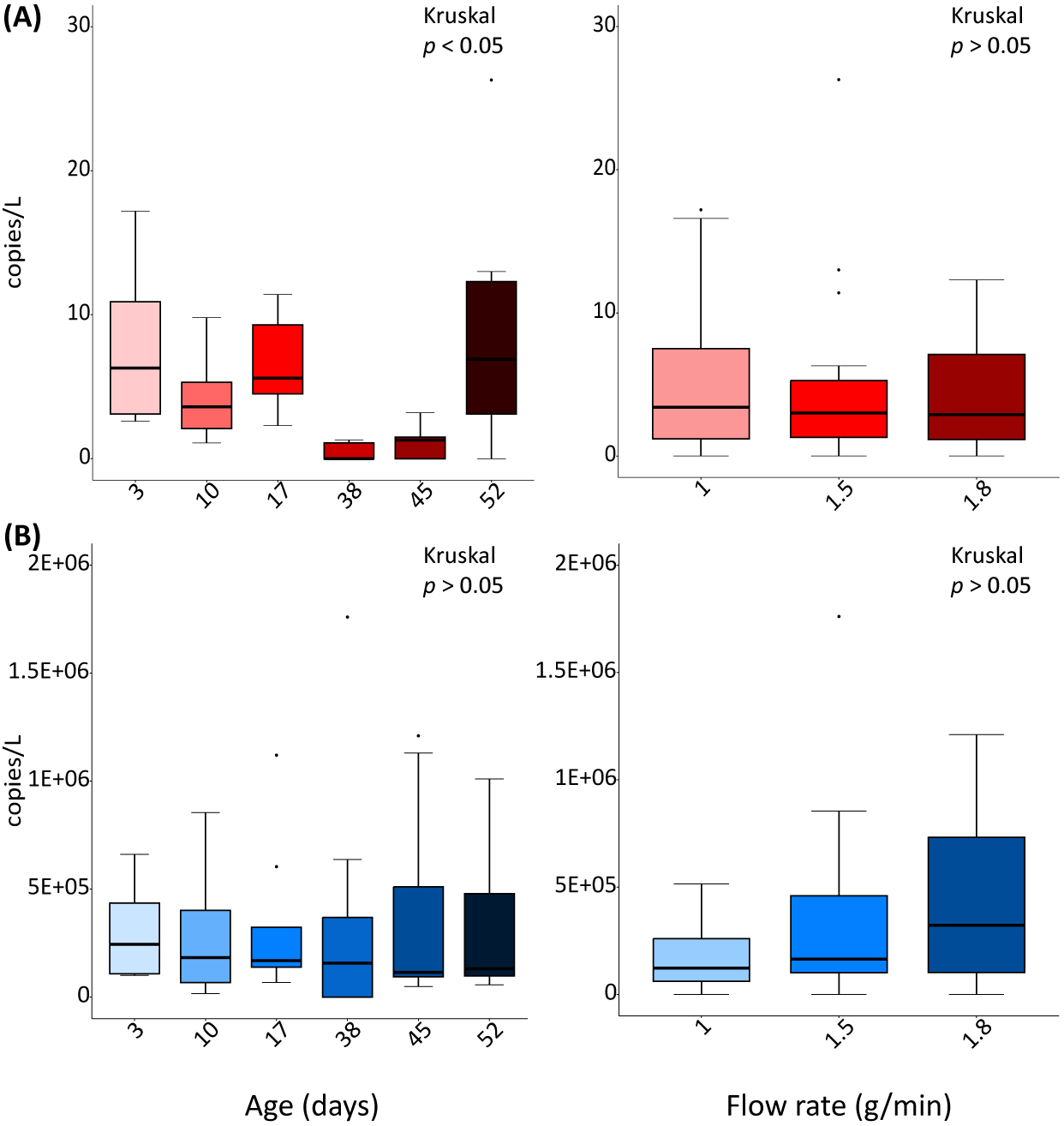


**Figure S2.** The concentrations of nontuberculous mycobacterium (NTM) in (A) air and (B) water samples quantified by droplet digital PCR.


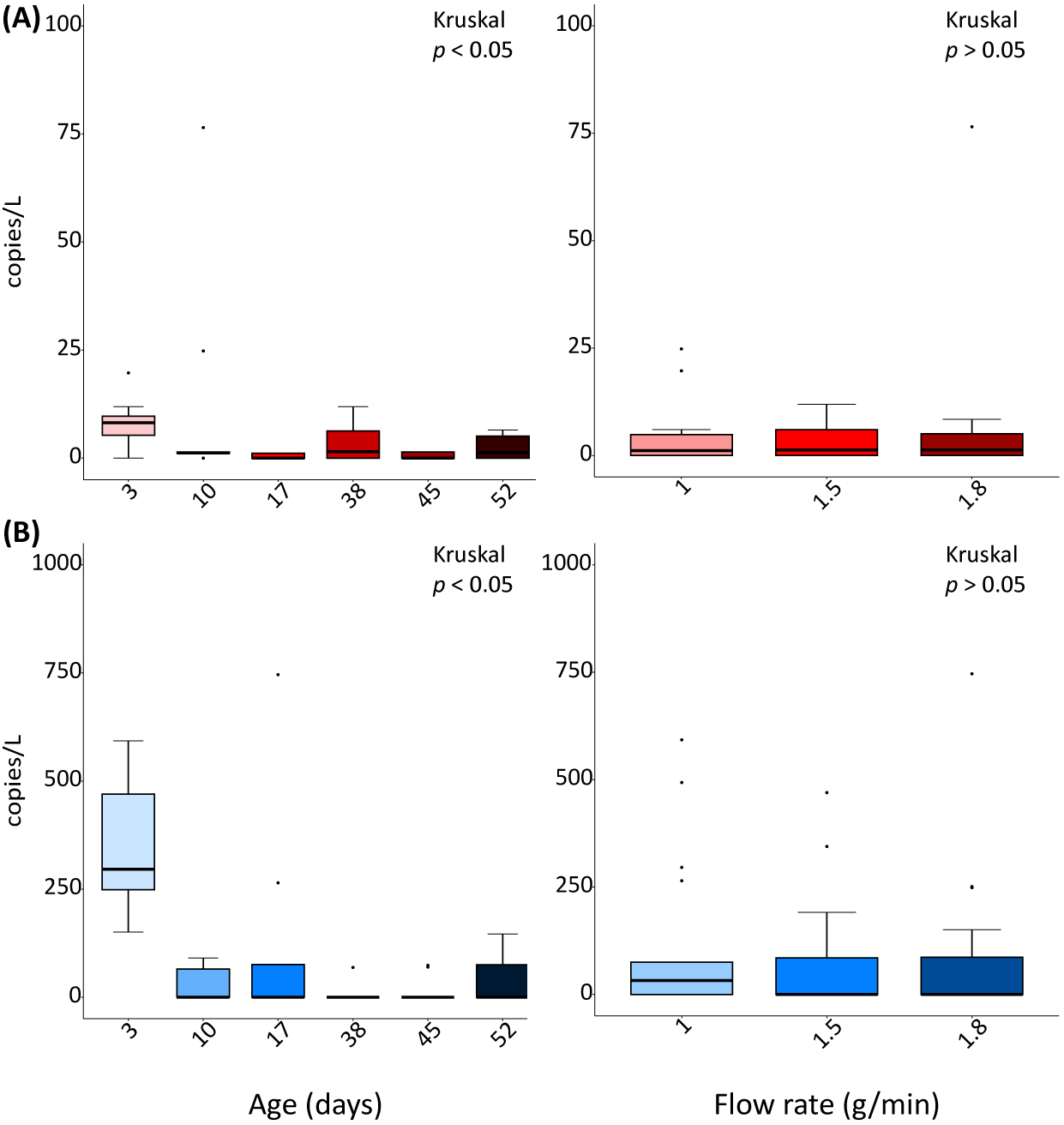


**Figure S3.** The concentrations of *Legionella pneumophila* in (A) air and (B) water samples quantified by droplet digital PC


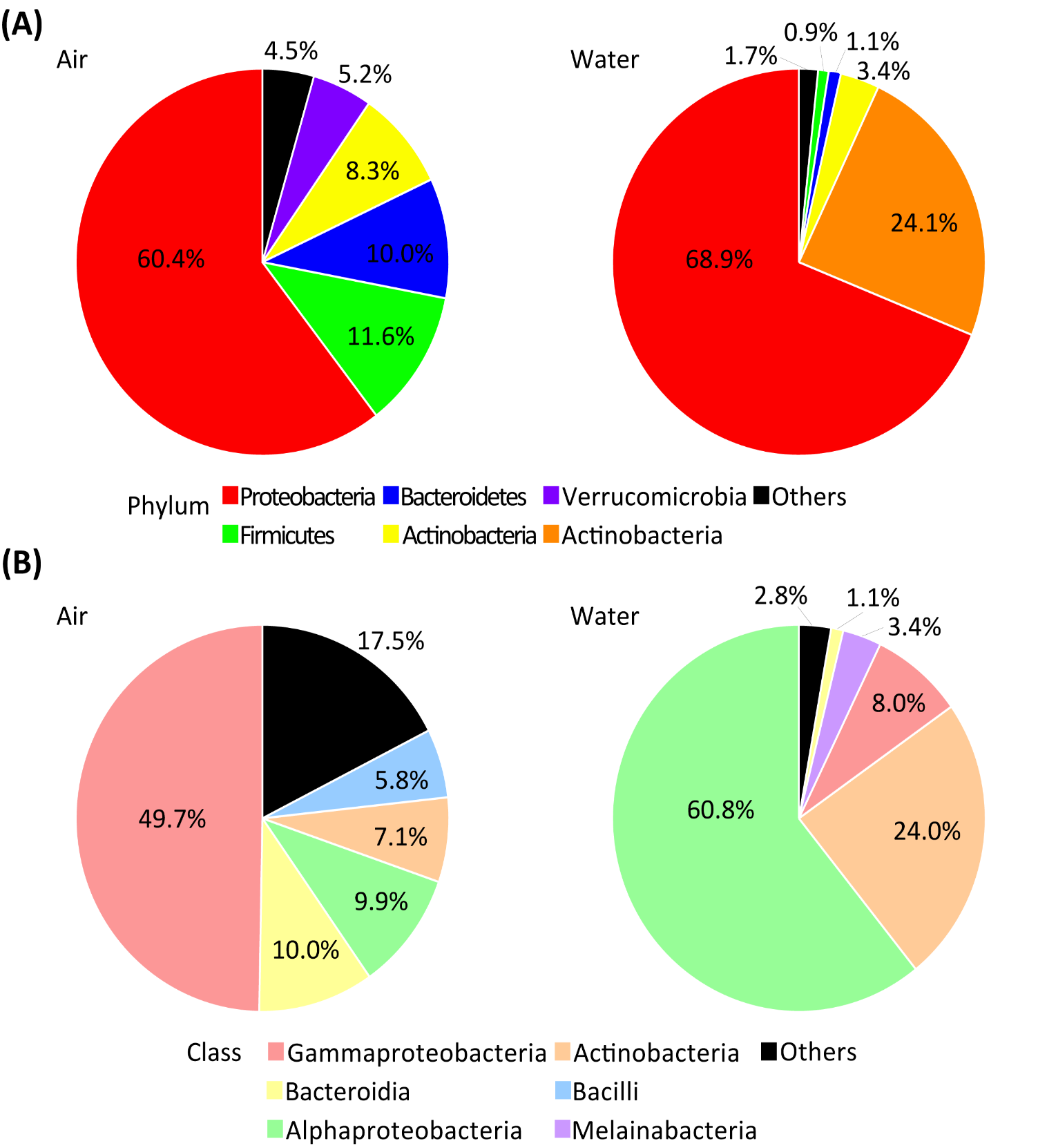
 **Figure S4.** Taxonomic composition of identified bacteria from each sample type in (A) phylum and (B) class level.
